# Detection of pediatric developmental delay with machine learning technologies

**DOI:** 10.1371/journal.pone.0324204

**Published:** 2025-05-20

**Authors:** Shin-Bo Chen, Chi-Hung Huang, Sheng-Chin Weng, Yen-Jen Oyang

**Affiliations:** 1 Graduate Institute of Biomedical Electronics and Bioinformatics, National Taiwan University, Taipei City, Taiwan (R.O.C.; 2 Department of Rehabilitation, En Chu Kong Hospital, New Taipei City, Taiwan (R.O.C.); 3 Department of Computer Science and Information Engineering, National Taiwan University, Taipei City, Taiwan (R.O.C.); Commonwealth Scientific and Industrial Research Organisation, AUSTRALIA

## Abstract

**Objective:**

Accurate identification of children who will develop delay (DD) is challenging for therapists because recent studies have reported that children who underwent early intervention achieved more favorable outcomes than those who did not. In this study, we have investigated how the frequencies of three types of therapy, namely the physical therapy, the occupational therapy, and the speech therapy, received by a child can be exploited to predict whether the child suffers from DD or not. The effectiveness of the proposed approach is of high interest as these features can be obtained with essentially no cost and therefore a prediction model built accordingly can be employed to screen the subjects who may develop DD before advanced and costly diagnoses are carried out.

**Methods:**

This study has been conducted based on a data set comprising the records of 2,552 outpatients (N = 34,862 visits, mean age = 72.34 months) collected at a hospital in Taiwan from 2012 to 2016. We then built 3 types of machine learning based prediction models, namely the deep neural network models (DNN), the support vector machine (SVM) models, and the decision tree (DT) models, to evaluate the effectiveness of the proposed approach.

**Results:**

Experimental results reveal that in terms of the F1 score, which is the harmonic mean of the sensitivity and the positive predictive value, the DT models outperformed the DNN models and the SVM models, if a high level of sensitivity is desired. In particular, the DT model developed in this study delivered the sensitivity at 0.902 and the positive predictive value at 0.723.

**Conclusions:**

What has been learned from this study is that the frequencies of the therapies that a child has received provide valuable information for predicting whether the child suffers from DD. Due to the performance observed in the experiments and the fact that these features can be obtained essentially without any cost, it is conceivable that the prediction models built accordingly can be wide exploited in clinical practices and significantly improve the treatment outcomes of the children who develop DD.

## Introduction

Developmental delay (DD) refers to a distinct set of early childhood developmental disabilities, and it is primarily diagnosed by assessing a child’s behavioral and mental capacities [1]. Rehabilitation physicians employ various methods, strategies, and diagnostic tools to diagnose and treat DD, including classification techniques. However, these classification methods are often subjective, time-consuming, and prone to inconclusive results. Moreover, they fail to clarify the underlying causes and are ineffective for early detection [2]. Early intervention significantly improves a child’s likelihood of reaching their full potential, with studies reporting that children who receive early intervention achieve more favorable outcomes than those who do not. Therefore, accurate classification of DD is crucial for providing effective early intervention services that ensure positive outcomes for children with DD [3].

Machine learning has been applied to develop novel computational methods that incorporate mathematical learning, statistical estimation, and information theories [4]. These methods automatically identify meaningful patterns within large datasets. A key advantage of machine learning is its ability to generate highly accurate and reliable predictions based on data comprising multiple variables. Additionally, machine learning enables causal inference from non-experimental datasets [5].

Psychiatric studies have successfully demonstrated that machine learning methods can be used to diagnose autism spectrum disorder (ASD) [6], classifying attention deficit hyperactivity disorder (ADHD) [7] based on altered event–related potentials, and identifying schizophrenia through free speech analysis [8]. For example, Bishop et al. employed machine learning to analyze the lifetime health issues of adults with ASD, accurately predicting cardiovascular, urinary, and respiratory conditions [9].

Studies have reported that that most cases of DD gradually resolve over time [10]. However, few studies have explored the application of machine learning methods to identify predictive factors and optimize rehabilitation therapy frequency for improved outcomes.

The novelty of our approach lies in utilizing therapy frequency as a predictive factor for DD classification. Machine learning methods enable valuable patterns to be automatically extracted from large data sets, and this capability is difficult to achieve with traditional statistical methods. This study proposes that machine learning can be used to identify key predictors of DD outcomes and thereby provide support for the development of personalized interventions and effective therapeutic strategies.

Researchers, including Osman Altay et al. [11], have used various classification methods to diagnose ASD, employing algorithms such as linear discriminant analysis (LDA) and K-nearest neighbors (K-NN). Notably, LDA has demonstrated higher precision compared to K-NN. Fatiha Nur, Ali Öztürk compared classification methods and reported achieving more favorable outcomes with the random forest method than with methods based on K-NN, naïve Bayes, and radial basis function (RBF) networks [12]. These literature findings underscore the importance of selecting appropriate algorithms in achieving accurate DD classification.

Integrating brain imaging data into machine learning models has produced promising results for enhancing DD classification. For example, Dvornek et al. combined phenotypic data with resting-state functional magnetic resonance imaging (rs-fMRI) and applied deep learning techniques for ASD classification [13]. Similarly, Liao et al. proposed a novel model based on community structure and deep learning, and they achieved improved accuracy with this model relative to that achieved using traditional methods [14]. Dekhil et al. integrated anatomical and functional information from structural MRI (sMRI) and functional MRI (fMRI), and they successfully applied their model to distinguish between autism and normal development [15]. These findings highlight the potential of incorporating brain imaging data to enhance DD classification.

Methods based on analyzing cortical measures and functional communication patterns have emerged as a valuable means of understanding DD. For instance, Yun Jiao et al. employed surface-based morphometry to classify ASD, revealing cortical thickness as a key predictive feature [16]. Heinsfeld et al. objectively classified patients with ASD by studying functional communication patterns derived from functional brain imaging and identifying relevant neural structures [17]. Research suggests that deep learning techniques can effectively differentiate individuals with ASD from those with typical development, reinforcing the utility of cortical measures and functional communication patterns in understanding DD.

Notable advances have been made in the development of algorithms for DD classification. Bone et al. developed highly effective, adaptable, and reliable algorithms for DD classification, outperforming existing methods. Their algorithms, which could weigh sensitivity and specificity separately, yielded promising results when they were used to analyze Autism Diagnostic Interview-Revised scores and Social Responsiveness Scale scores [18]. Jin Y et al. demonstrated the feasibility of using machine learning methods to classify infants at high risk of ASD at as early as 6 months after birth. Their multi-kernel support vector machine (SVM) classification system, which incorporated white matter tract and whole-brain integration features, exhibited improved accuracy relative to single-scale parameter networks [19]. A notable study by Kim et al. demonstrated the outstanding performance of an SVM system for predicting the prognosis of Class III malocclusion, with the system outperforming conventional statistical methods [20]. These advancements have led to the development of promising methods for enhancing early detection of and intervention strategies for DD.

Researchers have applied numerous methods for predicting DD. Table 1 presents a comprehensive summary of the existing machine learning based predictors for identifying patients who develop DD.

**Table 1:** A summary of the existing machine learning based predictors for identifying patients who may develop DD.

Classifier Used	Modality	Number of subjects	Features	Performance
RF, GBM [2]	MRI	876, including 417 (367 Males, 50 Females) ASD and 459 (382 Males, 77 Females) Typically Developing Children	White matter, Gray matter, cerebrospinal fluid, total intracranial volume	Accuracy = 60%
LDA, K-NN [11]	Questionnaire	292, including 141 ASD and 151 Non-ASD (4–11Yrs)	19 Different attribute/ Questions	AUC = 61%
NB, K-NN, RBFN, RF [12]	Questionnaire	244	21 Different attribute/ Questions	RF model: higher specificity of 96.4%
NN [13]	rsfMRI	1100, including 529 Autism and 571 typical controls	Phenotypic features, e.g., Age, Sex, Handedness, Full IQ, Eye status	DNN = 70.1%
Deep Learning [14]	rsfMRI	Total(ASD, NC) groupI: 38(19, 19); groupII: 110(55, 55); groupIII: 35(13, 22)	NMI matrix, Pearson matrix	NMI = 59.09% (D2 Dataset)
MDN [15]	sMRI, fMRI	47, including 22 autistic (20 Males, 2 Females) and 25 controls (all males))	Cerebral cortex, cerebral white Matter	Modality Fusion = 94.7%
SVM, FT, LMT [16]	MRI	38, including 22 ASD Children and 16 Normal Children	thicknesses, mean curvature, Gaussian curvature, folding index, curvature index	FT, LMT = 76%
DNN, SVM, RF [17]	rsfMRI, sMRI	1035, including 505 ASD individuals and 530 typical controls	Phenotypic features, e.g., Age, Sex, Handedness, Full IQ, Eye status	DNN = 70%
MLCV, SVM [18]	Questionnaire	1726, including 1264 ASD and 462 non-ASD	Correlation-based Features	ML Fusion = 89.2%
SVM [19]	MRI	80, including 40 High-Risk Infants (29 Males, 11 Females) and 40 Low-Risk Infants (27 Males,13 Female)	Multiscale Connectivity network of element	SVM = 76%

**Abbreviations:** linear discriminant analysis (LDA), mixture density network (MDN), naïve bayes (NB), k-nearest neighbor (KNN), radial basis function (RBF), gradient boosting model (GBM), normalized mutual information (NMI), resting-state functional magnetic resonance imaging (rsfMRI), structural MRI (sMRI), functional MRI (fMRI), deep neural network (DNN), random forest (RF), functional tree (FT), machine learning (ML), logistic model tree (LMT) and area under curve (AUC).

In summary, the studies addressed above primarily relied on DD symptoms to develop machine learning based prediction models. However, limitations pertaining to the accuracy and availability of the diagnostic data may impact the reliability of these machine learning based approaches [21–24]. In this study, we have proposed that the frequencies of three types of therapy, namely the physical therapy, the occupational therapy, and the speech therapy, received by a child can be exploited to predict whether the child suffers from DD or not. The effectiveness of the proposed approach is of high interest as these features can be obtained with high accuracy and essentially without any cost. Therefore, a prediction model built accordingly can be employed to screen the subjects who may develop DD before advanced and costly diagnoses are carried out. Due to the performance observed, it is anticipated that the proposed prediction models can be wide exploited in clinical practices and significantly improve the treatment outcomes of the children who develop DD.

## Methods

### Data collection and outcome measurement

In the present study, all patients included in the clinical group were previously given a diagnosis based on the criteria established in the *Diagnostic and Statistical Manual of Mental Disorders-V-TR (DSM-5-TR)* [25–26]. For example, the DSM-5-TR defines autism spectrum disorder (ASD) as involving persistent deficits in social communication across multiple environments, as outlined in the relevant diagnostic criterion. Assessments of comorbid psychiatric diagnoses and development of treatment plans were completed by child psychiatrists. The main caregivers of the included participants received assistance from rehabilitation therapists with gathering sociodemographicand rehabilitation clinical information and completing several forms.

**Fig 1:**
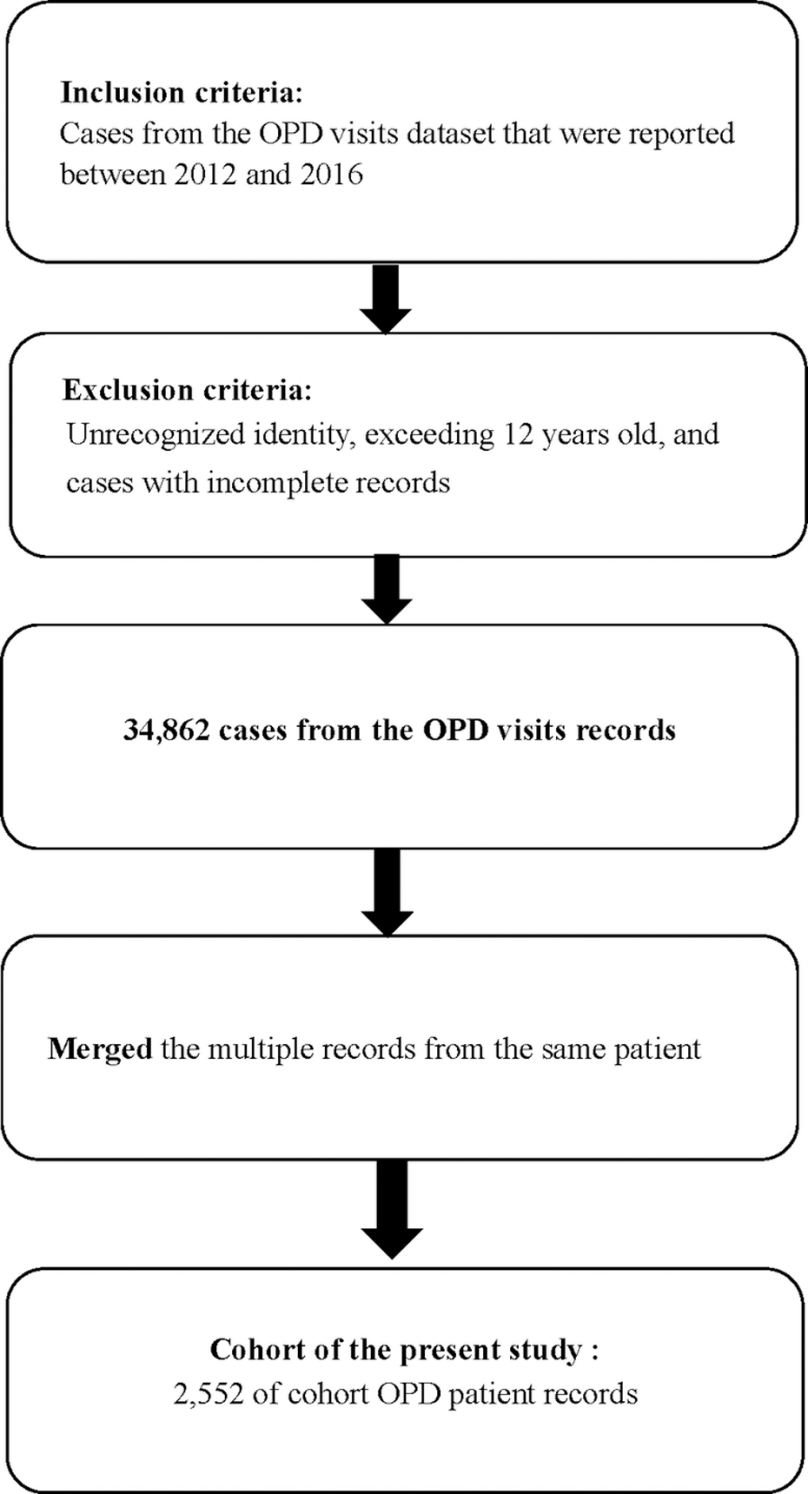
Flow diagram for generating the study dataset.

Assessments of DD symptoms were conducted by a rehabilitation physician who used the Rehabilitation Developmental Evaluation Form. In the outpatient department (OPD) of the study hospital, children with DD or child and adolescent psychiatry patients typically received rehabilitation therapy, and a data form was used to update their medical service records, which included information pertaining to the frequencies of occupational therapy service (OTS), physical therapy service (PTS), and speech therapy service (STS). The dataset used in this study comprises the medical records of the outpatients who visited the rehabilitation clinic of the study hospital with suspected DD between January 1, 2012, and December 31, 2016. The Institutional Review Board of the En Chu Kong Hospital reviewed the above documents and approved the study on 2024/07/23 (ECK-IRB Number: ECKIRB1130501). This approval is valid till 2025/07/22. To protect patient information and confidentiality, no subject names were collected. Each patient was anonymously assigned a study ID. Supporting information S1 Table provides the *International Classification of Diseases, 9th Revision, Clinical Modification* (*ICD-9-CM*) codes used to define DD. The patients’ records extracted from the dataset included age, gender, and the frequencies of OTS, PTS, and STS received. Specific DD problems and disabilities were determined using a comprehensive literature review and after a consensus was reached by rehabilitation physicians and child psychiatry specialists. *The International Classification of Diseases, 10th Revision, Clinical Modification (ICD-10-CM)* codes used to identify various types of DD are presented in Supporting information Table S2. Fig 1 illustrates the participant selection process as a flow diagram. This study identified 2552 outpatients with age under 12 years who made one or more OPD visits. Among these patients, 1719 (67.4%) had DD. The total number of OPD visits was 34,862. Table 2 presents the demographic and clinical characteristics of the patients with DD. It is observed that due to the strict flowchart employed by the hospital, the outpatient medical records from which our dataset was derived are highly accurate and include minimal missing data and few unmeasured confounding variables.

**Table 2:** Demographic and clinical characteristics of patients with DD (n = 2552).

Characteristics	Category	No. (%)	Chi-squared test P value
Developmental Delay (DD)	**Yes**	**1719 (67.4)**	
	No	833 (32.6)	
Gender	**Men**	**1778 (69.6)**	**P < 0.001** ^***^
	Women	774 (30.4)	
Age in months (Mean ± SD)		**60.5** ± **30.7**	
OTS	**Yes**	**2152 (84.3)**	**P < 0.001** ^***^
	No	400 (15.7)	
PTS	**Yes**	**1624 (63.6)**	**P < 0.001** ^***^
	No	928 (36.3)	
STS	**Yes**	**1570 (61.5)**	
	No	982 (38.5)	

**NOTE.** The p-values were calculated based on the χ2 test of independence.

**Abbreviations**: standard deviation (SD).

The present study was approved by the ethics committee of En Chu Kong Hospital prior to data collection. Informed consent was waived by the committee because of the de-identification and non-interventional design of the present study.

### Experimental procedures

**Fig 2:**
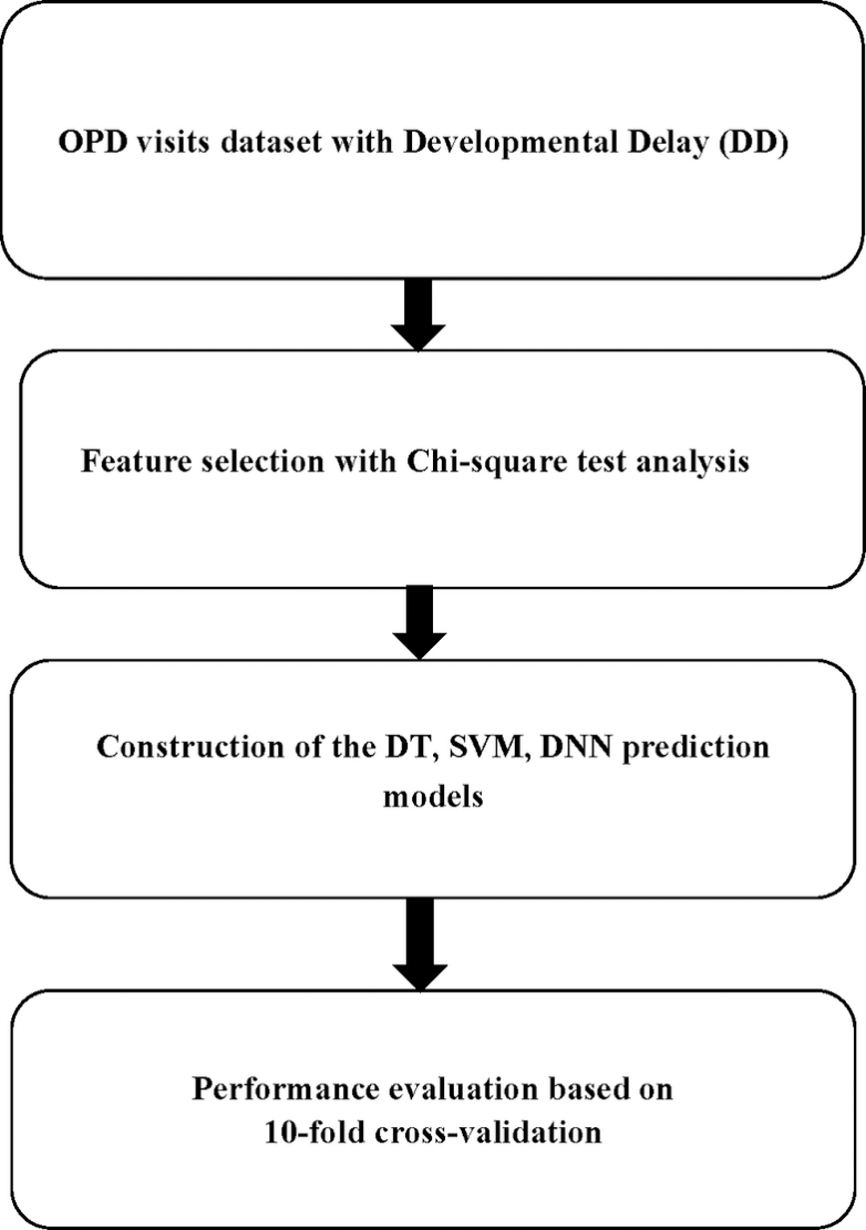
The experimental procedure.

The present study extracted information from OPD records, including data on demographic characteristics, such as gender, age, and the frequencies of therapy services used (OTS, PTS and STS). The patients were divided into two groups, namely a DD group and a non-DD group. Fig 2 presents the experimental procedure that was employed to assess the performance of several prediction models.

### Feature selection

In this study, we included 4 features in our dataset: gender and frequencies of OTS, PTS, and STS. In this respect, the frequency of a particular therapy service was defined to be the average number of services received by a patient in one year. Then, we conducted chi-squared tests to figure out whether a feature was correlated to the outcome variable [27–29]. For feature gender, we carried out the chi-squared test of independence. For the frequencies of OTS, PTS, and STS, we carried out the chi-squared test of goodness of fit, with the null hypothesis set to the average frequency of patients with DD equal to the average frequency of patients without DD [30]. Table 3 shows the p-values obtained from these tests. Accordingly, we included gender and frequencies of OTS, PTS, and STS to build the prediction models.

**Table 3:** Feature selection with the Chi-square Test.

Variables	P value
Gender	0.000022
OTS	0.000000
PTS	0.000000
STS	0.000000

### Development of prediction models and performance evaluation

In this study, we investigated the prediction performance of three categories of machine learning models, namely the DT models [31–33], the SVM models [20], and the DNN models [34]^.^ The DT models are preferred by many clinicians due to the explicit decision rules output by the algorithm. On the other hand, the SVM models and the DNN models are two categories of the most advanced machine learning models that can generally outperform the DT models due to the non-linear transformations invoked in the prediction process. However, the non-linear transformations invoked also make it almost impossible for a user to comprehend how the prediction is made. As a result, many clinicians are reluctant to trust the models that work like a black box. Therefore, it is of interest to investigate how the performance of alternative categories of machine learning models compares. If the performance of the DT models observed in the experiments is comparable with the performance of the advanced machine learning models, which was observed in our recent studies [35,36], then the DT models are favorite due to explicit decision rules output by the algorithm.

In order to obtain comprehensive pictures of how each category of prediction models performed, we employed alternative parameter settings to generate prediction models with different performance characteristics. Table 4 provides a summary of the software packages and alternative parameter settings employed to build the prediction models. Then, we conducted 10-fold cross validation to evaluate the performance characteristics of each prediction model generated [37–39]. The performance metrics considered in this study include accuracy, sensitivity, specificity, positive predictive value (PPV as known as precision), and F1 score. The F1 score, which is the harmonic mean of the sensitivity and the PPV, is commonly employed in machine learning research and has increasingly been employed in biomedical research [40]. Furthermore, for each category of prediction models, e.g., the DT models, the SVM models, or the DNN models, we evaluated its overall performance based on the area under the receiver operating characteristic (ROC) curve. In order to generate the ROC curve, we picked up the prediction model that delivered the highest F1 score at each level of sensitivity.

**Table 4:** Software packages and parameter settings employed to build the models.

Model	Programming Language	Package	Parameters
DT	Python	pandas sklearn	split = “information”, prior = 0.01 ~ 0.9 with 0.008 step size, cp=[0.05,0.04,0.03,0.02,0.01]
DNN	Python	Tensor Flow	Input neurons=[4] for 4 features set, Hidden neurons=[5,10,27] Hidden layer=[3,5,9]
SVM	Python	sklearn svm	kernel=[‘linear’,’rbf’,’poly’], C=[0.01,0.1,1,3,10,15,20], gamma = ‘auto’, probability = True Best for rbf-kernal (cost = 10, gamma = 0.25, epsilon = 0.1)

## Results

Fig 4 shows ROC curves [41,42] and the corresponding areas under the curves (AUCs) of the DT, SVM, and DNN models. Table 5 shows the detailed performance data of the models that delivered sensitivities at the 0.80 level and at the 0.90 level. It is observed that the DNN models and the DT models outperformed the SVM models in terms of AUC. On the other hand, as shown in Table 5, if a high level of sensitivity is desirable, then the DT models significantly outperformed the DNN models and the SVM models in terms of the F1 score, which is the harmonic mean of the sensitivity (also called recall) and the positive predictive value (PPV, also called precision).

**Fig 3:**
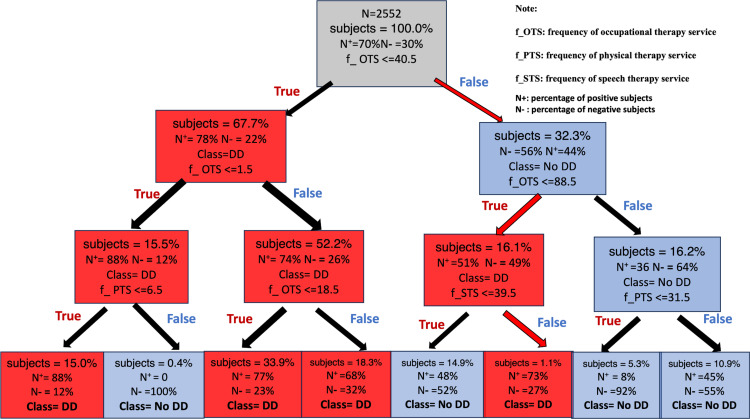
The structure of the DT model generated by feeding our dataset into the software package and with cp and prior set to 0.01 and 0.55, respectively.

**Table 5:** Detailed performance characteristics of alternative prediction models.

Target Sensitivity	Model	Accuracy	Sensitivity	Specificity	Precision	F1 Score
0.8	DT	0.650	0.802	0.337	0.734	0.744
	SVM	0.597	0.802	0.488	0.454	0.580
	DNN	0.663	0.808	0.587	0.509	0.624
0.9	DT	0.701	0.902	0.289	0.723	0.803
	SVM	0.487	0.898	0.269	0.395	0.548
	DNN	0.616	0.904	0.464	0.472	0.620

**Fig 4:**
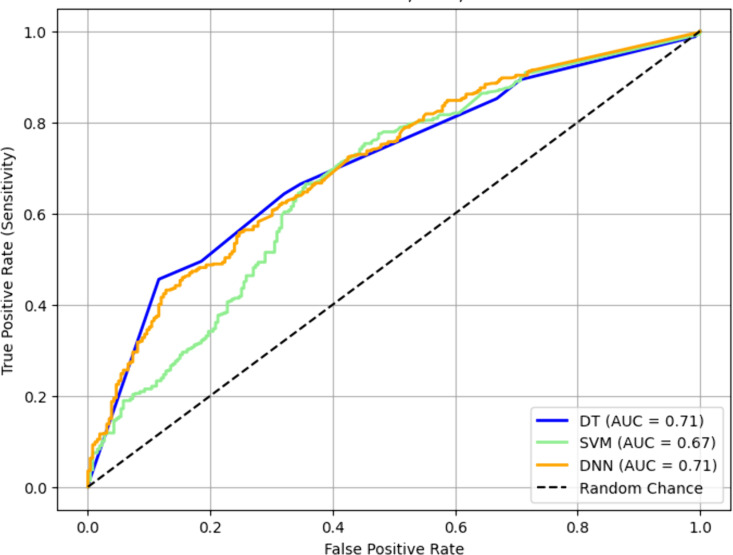
ROC curves of the DNN, DT, SVM models.

Based on the data shown in Table 5, it is conceivable that the DT model that delivered the sensitivity at the 0.90 level is the favorite choice due to two reasons. Firstly, the PPV with this particular DT model is significantly higher than the PPVs with the SVM model and the DNN model that delivered the same level of sensitivity. Therefore, in clinical applications, the number of false positive predicted by this DT model should be significantly lower than the numbers of false positive predicted by the SVM model and the DNN model with the same level of sensitivity. Secondly, the PPV with this DT model is almost the same as the PPV with the DT model that delivered the sensitivity at the 0.80 level. Accordingly, in the subsequent discussions, we will focus on the DT model that delivered the sensitivity at the 0.90 level.

Fig 3 shows the structure of the DT model generated by feeding our dataset into the software package and with cp and prior set to 0.01 and 0.55 respectively. According to our performance evaluation, this DT model should be able to deliver a sensitivity at the 0.90 level and a PPV above 0.70. The top-down path, following the red arrows, illustrates how the prediction for a subject with sex = female, f_OTS = 60, f_PTS = 30, and f_STS = 50 is made. The prediction made is positive, i.e., the subject suffers from DD, as the path ends at a red node. On the other hand, a subject is predicted to be negative, if the path corresponding to the subject’s feature values ends at a blue node. The n+ and n- values associated with each node respectively specify the percentages of positive subjects and negative subjects among all the subjects that meet the criteria specified along the path to the node. In fact, a user can figure out the probability that a subject is positive or negative by examining the n+ and n- of the leaf node that the feature values of this subject fit into.

## Discussion

In this study, we have investigated how the frequencies of therapies can be exploited to build machine learning based prediction models for identifying children with development delay. Based on the experimental results observed, it is conceivable that the proposed approach can be widely exploited in clinical practices due to several reasons. Firstly, the performance observed with the prediction models developed in this study should meet the criteria acceptable by most physicians. For example, based on our experimental results, we can anticipate that the DT model shown in Table 5 can identify about 90.0% of the subjects who will develop DD in the future, while about 72% of the subjects predicted to be positive are actually true positives. Secondly, the features employed to build the prediction models can be obtained with essentially no costs. Therefore, the prediction models can be exploited to screen the subjects who may develop DD before advanced and costly diagnoses are carried out.

The experimental results also demonstrate that for the applications targeted by this study we do not need to trade performance for the interpretability of the prediction model. The F1 scores presented in Table 5 show that the DT models that delivered the sensitivity at the 0.90 level and at the 0.80 level outperformed the DNN models and the SVM model that delivered the sensitivity at the same level. For most applications, it is typical that advanced machine learning based prediction models such as the DNN models and the SVM models outperform the DT models due to the non-linear transformations invoked. However, the non-linear transformations invoked also make it almost impossible for a user to figure out how the prediction is made. Fortunately, for our applications, we do not need to trade performance for the interpretability of the prediction model.

The DT structure shown in Fig 3 illustrates how a user can examine the structure to figure out the decision rules followed by the prediction model to make predictions. Furthermore, the ratio of between the number of positive subjects and the number of negative subjects at each leaf node specifies how likely a subject that meets the criteria corresponding to the path to this particular leaf node develops DD. For example, the probability that the subject with sex = female, f_OTS = 60, f_PTS = 30, and f_STS = 50 develops DD is 0.73. In clinical practice, a physician can refer to this specific probability and his/her clinical experiences to make the final diagnosis.

In summary, the major finding due to this study is that the frequencies of the therapies that a child has received provide valuable information for predicting whether the child suffers from DD. Due to the performance observed in the experiments and the fact that these features can be obtained essentially without any cost, it is conceivable that the prediction models built accordingly can be wide exploited in clinical practices and significantly improve the treatment outcomes of the children who develop DD. Though the study was based on a dataset collected in a hospital in Taiwan, we anticipate that the proposed method can be exploited to build accurate prediction models for populations in different countries with various race groups.

### Limitations

Several limitations of this study should be noted. Firstly, this retrospective study relies on data extracted from the outpatient (OPD) database with children under 12 years old. Consequently, the findings may not be generalized for the other age groups. Secondly, the prediction models developed were solely based on the data collected from a hospital in Taiwan and its applicability to other hospitals has not been validated. Thirdly, the dataset employed in this study was derived from the clinical records in the OPD and therefore these patients were likely to already have DD conditions. Finally, it is observed that there were significantly more male patients than the female patients, which conforms with previous findings [43,44]. Therefore, stratified sampling based on gender was not carried out.

## Conclusions and future works

As this study has revealed that the frequencies of the therapies that a child has received provide valuable information for predicting whether the child suffers from DD, it is conceivable that we can built more accurate prediction models by integrating these features with other clinical assessment scores and the records of advanced medical examinations such as brain imaging, electroencephalograms, etc. This study has also revealed that concerning this application the performance delivered by the DT models is favorite in comparison with that delivered by advanced machine learning models such as the SVM models and the DNN models. As the DT structures explicitly exhibit the decision rules employed to make predictions, physicians can incorporate these decision rules with their clinical experiences to make final diagnoses. For future studies, it is of interest to investigate the association between a child’s age and the frequency of clinical therapy. In particular, it is of interest to investigate whether the therapy frequency peaks within a specific age range. The findings may support the principle of early intervention and carry significant clinical and therapeutic implications. Furthermore, identifying the age at which a child with DD is diagnosed, the specific clinical therapy received within rehabilitation, and the period of highest therapy concentration can facilitate our understanding of the most critical timeframe for early intervention.

## Supporting information

S1 Table The ICD-9-CM (International Classification of Disease, 9th Revision, Clinical Modification) codes of developmental delay (DD). (DOCX)

S2 Table The ICD-10-CM (International Classification of Disease, 10th Revision, Clinical Modification) codes of developmental delay (DD). (DOCX)

## Abbreviations

DD: developmental delay
ASD: autism spectrum disorder
DT: decision tree
SVM: support vector machine
DNN: deep neural network
OTS: occupational therapy service
PTS: physical therapy service
STS: speech therapy service
OPD: outpatient department
