# Supplementary material for: Detection of pediatric developmental delay with machine learning technologies
Source: PLoS One. 2025 May 20;20(5):e0324204. doi: 10.1371/journal.pone.0324204 (PMC12091767; doi:10.1371/journal.pone.0324204)
Supplement: S1 Table — (DOCX) [file pone.0324204.s001.docx]

**Supporting information**

**[Table S1](#_Methods)** The ICD-9-CM (International Classification of Disease, 9th Revision, Clinical Modification) codes of developmental delay (DD)

| ICD-9-CM | Description | | | | | | | | |
| --- | --- | --- | --- | --- | --- | --- | --- | --- | --- |
| 191 | Malignant neoplasm of cerebrum, except lobes and ventricles | | | | | |  |  |  |
| 228 | Hemangioma of unspecified site | | | |  |  |  |  |  |
| 229 | Benign neoplasm of lymph nodes | | | |  |  |  |  |  |
| 250 | Diabetes Mellitus | |  |  |  |  |  |  |  |
| 270 | Disturbances of amino acid transport | | | |  |  |  |  |  |
| 277 | Other and unspecified disorders of metabolism | | | | |  |  |  |  |
| 296 | Manic disorder, single episode, unspecified | | | | |  |  |  |  |
| 299 | Pervasive developmental disorders | | | |  |  |  |  |  |
| 300 | Anxiety, dissociative and somatoform disorders | | | | |  |  |  |  |
| 307 | Special symptoms or syndromes not elsewhere classified | | | | | |  |  |  |
| 313 | Disturbance of emotions specific to childhood and adolescence | | | | | | |  |  |
| 314 | Hyperkinetic syndrome of childhood | | | |  |  |  |  |  |
| 315 | Specific delays in development | | | |  |  |  |  |  |
| 315.9 | Unspecified delay in development | | | |  |  |  |  |  |
| 317 | Mild intellectual disabilities | | |  |  |  |  |  |  |
| 318 | Other specified mental retardation | | | |  |  |  |  |  |
| 319 | Unspecified intellectual disabilities | | | |  |  |  |  |  |
| 331 | Other cerebral degenerations | | |  |  |  |  |  |  |
| 335 | Anterior horn cell disease | | |  |  |  |  |  |  |
| 336 | Other diseases of spinal cord | | |  |  |  |  |  |  |
| 343 | Infantile cerebral palsy | | |  |  |  |  |  |  |
| 345 | Epilepsy and recurrent seizures | | | |  |  |  |  |  |
| 348 | Other conditions of brain | | |  |  |  |  |  |  |
| 351 | Facial nerve disorders | | |  |  |  |  |  |  |
| 353 | Nerve root and plexus disorders | | | |  |  |  |  |  |
| 359 | Muscular dystrophies and other myopathies | | | | |  |  |  |  |
| 360 | Disorders of the globe | | |  |  |  |  |  |  |
| 372 | Disorders of conjunctiva | | |  |  |  |  |  |  |
| 389 | Hearing loss | |  |  |  |  |  |  |  |
| 431 | Intracerebral hemorrhage | | |  |  |  |  |  |  |
| 438 | Late effects of cerebrovascular disease | | | |  |  |  |  |  |
| 446 | Polyarteritis nodosa and allied conditions | | | | |  |  |  |  |
| 485 | Bronchopneumonia organism unspecified | | | | |  |  |  |  |
| 493 | Asthma |  |  |  |  |  |  |  |  |
| 521 | Diseases of hard tissues of teeth | | | |  |  |  |  |  |
| 536 | Disorders of function of stomach | | | |  |  |  |  |  |
| 550 | Inguinal hernia | |  |  |  |  |  |  |  |
| 691 | Atopic dermatitis and related conditions | | | |  |  |  |  |  |
| 718 | Other derangement of joint | | |  |  |  |  |  |  |
| 719 | Other and unspecified disorders of joint | | | |  |  |  |  |  |
| 723 | Other disorders of cervical region | | | |  |  |  |  |  |
| 724 | Other and unspecified disorders of back | | | |  |  |  |  |  |
| 728 | Disorders of muscle ligament and fascia | | | |  |  |  |  |  |
| 734 | Flat foot |  |  |  |  |  |  |  |  |
| 736 | Other acquired deformities of limbs | | | |  |  |  |  |  |
| 737 | Curvature of spine | |  |  |  |  |  |  |  |
| 738 | Other acquired musculoskeletal deformity | | | | |  |  |  |  |
| 742 | Other congenital anomalies of nervous system | | | | |  |  |  |  |
| 743 | Congenital anomalies of eye | | |  |  |  |  |  |  |
| 745 | Bulbus cordis anomalies and anomalies of cardiac septal closure | | | | | | |  |  |
| 749 | Cleft palate and cleft lip | | |  |  |  |  |  |  |
| 754 | Certain congenital musculoskeletal deformities | | | | |  |  |  |  |
| 756 | Other congenital musculoskeletal anomalies | | | | |  |  |  |  |
| 758 | Chromosomal anomalies | | |  |  |  |  |  |  |
| 759 | Other and unspecified congenital anomalies | | | | |  |  |  |  |
| 765 | Disorders relating to short gestation and unspecified low birthweight | | | | | | |  |  |
| 780 | General symptoms | |  |  |  |  |  |  |  |
| 781 | Symptoms involving nervous and musculoskeletal systems | | | | | |  |  |  |
| 783 | Symptoms concerning nutrition metabolism and development | | | | | | |  |  |
| 784 | Symptoms involving head and neck | | | |  |  |  |  |  |
| 799 | Other ill |  |  |  |  |  |  |  |  |
| 813 | Fracture of radius and ulna | | |  |  |  |  |  |  |
| 816 | Fracture of one or more phalanges of hand | | | | |  |  |  |  |
| 827 | Other multiple and ill | | |  |  |  |  |  |  |
| 848 | Other and ill | |  |  |  |  |  |  |  |
| 852 | Subarachnoid subdural and extradural hemorrhage following injury | | | | | | |  |  |
| 854 | Intracranial injury of other and unspecified nature | | | | |  |  |  |  |
| 943 | Burn of upper limb except wrist and hand | | | | |  |  |  |  |
| 959 | Injury other and unspecified |  |  |  |  |  |  |  |  |
