# Supplementary material for: Detection of pediatric developmental delay with machine learning technologies
Source: PLoS One. 2025 May 20;20(5):e0324204. doi: 10.1371/journal.pone.0324204 (PMC12091767; doi:10.1371/journal.pone.0324204)
Supplement: S2 Table — (DOCX) [file pone.0324204.s002.docx]

**Supporting information**

**Table S2** The ICD-10-CM (International Classification of Disease, 10th Revision, Clinical Modification) codes of developmental delay (DD)

| ICD-10-CM | | Description | | | | | | | | |
| --- | --- | --- | --- | --- | --- | --- | --- | --- | --- | --- |
| D82 | Immunodeficiency associated with other major defects | | | | | | |  |  |  |
| F70 | Mild intellectual disabilities | | | |  |  |  |  |  |  |
| F78 | Other intellectual disabilities | | | |  |  |  |  |  |  |
| F80 | Specific developmental disorders of speech and language | | | | | | |  |  |  |
| F81 | Specific reading disorder | | | |  |  |  |  |  |  |
| F82 | Specific developmental disorder of motor function | | | | | |  |  |  |  |
| F84 | Pervasive developmental disorders | | | | |  |  |  |  |  |
| F88 | Other disorders of psychological development | | | | | |  |  |  |  |
| F89 | Unspecified disorder of psychological development F89 | | | | | | |  |  |  |
| F90 | Attention | | deficit hyperactivity disorders | | |  |  |  |  |  |
| F94 | Disorders of social functioning with onset specific to childhood and adolescence | | | | | | | |  |  |
| F98 | Other behavioral and emotional disorders with onset usually occurring in childhood and adolescence | | | | | | | |  |  |
| G40 | Epilepsy and recurrent seizures | | | | |  |  |  |  |  |
| G80 | Cerebral palsy | | |  |  |  |  |  |  |  |
| G93 | Other disorders of brain | | | |  |  |  |  |  |  |
| I62 | Other and unspecified nontraumatic intracranial hemorrhage | | | | | | |  |  |  |
| J06 | Acute upper respiratory infections of multiple and unspecified sites | | | | | | | |  |  |
| M20 | Acquired deformities of fingers and toes | | | | |  |  |  |  |  |
| M21 | Other acquired deformities of limbs | | | | |  |  |  |  |  |
| M41 | Scoliosis | |  |  |  |  |  |  |  |  |
| M43 | Other deforming dorsopathies | | | | |  |  |  |  |  |
| M65 | Synovitis and tenosynovitis | | | |  |  |  |  |  |  |
| P07 | Disorders of newborn related to short gestation and low birth weight, not elsewhere classified | | | | | | | |  |  |
| Q21 | Congenital malformations of cardiac septa | | | | | |  |  |  |  |
| Q65 | Congenital deformities of hip | | | |  |  |  |  |  |  |
| Q87 | Other specified congenital malformation syndromes affecting multiple systems | | | | | | | | |  |
| R09 | Other symptoms and signs involving the circulatory and respiratory system | | | | | | | | |  |
| R26 | Abnormalities of gait and mobility | | | | |  |  |  |  |  |
| R27 | Other lack of coordination | | | |  |  |  |  |  |  |
| R41 | Other symptoms and signs involving cognitive functions and awareness | | | | | | | |  |  |
| R45 | Symptoms and signs involving emotional state | | | | | |  |  |  |  |
| R47 | Speech disturbances, not elsewhere classified | | | | | |  |  |  |  |
| R56 | Convulsions, not elsewhere classified | | | | |  |  |  |  |  |
| R62 | Lack of expected normal physiological development in childhood and adults | | | | | | | | |  |
| S40 | Superficial injury of shoulder and upper arm | | | | | |  |  |  |  |
| S90 | Superficial injury of ankle, foot and toes | | | | |  |  |  |  |  |
| T14 | Injury of unspecified body region | | | | |  |  |  |  |  |
| V06 | Pedestrian injured in collision with other nonmotor vehicle | | | | | | |  |  |  |
| V20 | Motorcycle rider injured in collision with pedestrian or animal | | | | | | | |  |  |
| Z23 | Encounter for immunization | | | |  |  |  |  |  |  |
