# Supplementary material for: Detection of pediatric developmental delay with machine learning technologies
Source: PLoS One. 2025 May 20;20(5):e0324204. doi: 10.1371/journal.pone.0324204 (PMC12091767; doi:10.1371/journal.pone.0324204)
Supplement: Attachment — Submitted filename: Response to Reviewers.docx [file pone.0324204.s004.docx]

**Comment 1 from Reviewer #1:**

Thanks for the opportunity to review the current study, which described using machine learning technologies to identify patients with developmental delay. This is an important area that is worthwhile for further research. The study has a particular strength in using a large sample size in real-life settings for the model development.

The study is generally well-written. It covered key concepts in in the introduction. It has also provided a comprehensive summary of previous literatures.

However, there is two major concerns that affect the paper's contribution in its current state.

Comment 1: In the discussion section, there is a need to discuss the results in more details, e.g., the use of different models (SVM, RBF, DNN, etc.). Currently, there are lots of important aspects of the findings have not been well discussed. Therefore, it makes the limitation, future research directions, and conclusion not strongly connected and supported by the current findings.
Comment 2: Whereas the authors mentioned sensitivity and accuracy in the results section, the authors have more focus on reporting and discussing sensitivity. It could be important to discuss specificity in more details in the both results and discussion section. Specificity is also an key factors determining overall accuracy. Particularly, in the results section, the authors reported, 'DT model achieved a higher accuracy of up to 72%', 'DNN model achieved a higher sensitivity of up to 95% (accuracy = 0.70, F1 score = 0.81).', 'RBF kernel can identify problems with an accuracy of up to 71% (sensitivity = 0.93, F1 score = 0.81)' However, RBF actually has a very low specificity of 0.233. DT maintains a relatively higher specificity of o.61, and DNN has a very low specificity of 0.19. These information are also very important in determining the accuracy of the model and suggest to be further considered and discussed further.

**Responses:**

1. We have revised the “4” section significantly. Please refer to the part from line 270 on page 18 to line 297 on page 19. We have also extracted this part and attached it here.

“In this study, we have investigated how the frequencies of therapies can be exploited to build machine learning based prediction models for identifying children with development delay. Based on the experimental results observed, it is conceivable that the proposed approach can be widely exploited in clinical practices due to several reasons. Firstly, the performance observed with the prediction models developed in this study should meet the criteria acceptable by most physicians. For example, based on our experimental results, we can anticipate that the DT model shown in Table 5 can identify about 90.0% of the subjects who will suffer from DD in the future, while about 72% of the subjects predicted to be positive are true positives. Secondly, the features employed to build the prediction models can be obtained with essentially no costs. Therefore, the prediction models can be exploited to screen the subjects who may suffer from DD before advanced and costly diagnoses are carried out.

The experimental results also demonstrate that for the applications targeted by this study we do not need to trade performance for the interpretability of the prediction model. The F1 scores presented in Table 5 show that the DT models that delivered the sensitivity at the 0.90 level and at the 0.80 level outperformed the DNN models and the SVM model that delivered the sensitivity at the same level. For most applications, it is typical that advanced machine learning based prediction models such as the DNN models and the SVM models outperform the DT models due to the non-linear transformations invoked. However, the non-linear transformations invoked also make it almost impossible for a user to figure out how the prediction is made.

The DT structure shown in Fig3 illustrates how a user can examine the structure to figure out the decision rules followed by the prediction model to make predictions. Furthermore, the ratio of between the number of positive subjects and the number of negative subjects at each leaf node specifies how likely a subject that meets the criteria corresponding to the path to this particular leaf node suffers from DD. For example, the probability for the subject illustrated in Fig 3 to suffer from DD is 0.727. In clinical practice, a physician can refer to this specific probability and his/her clinical experiences to make the final diagnosis.”

2. In the revised manuscript, we have added a figure that shows the ROC curves of the 3 types of prediction models and provided the corresponding AUCs. Please refer to Fig 4. The ROC curves provide the users with overall pictures of how these different types of prediction modes performed with different parameter settings. Considering clinical applications, we speculate that physicians may be more interested in the prediction models that can deliver a high level of sensitivity. Therefore, in our revised manuscript, we added Table 5 on page 18, which shows the detailed performance data of the prediction models that deliver the sensitivity at the 0.80 and 0.90 levels and is attached here. Furthermore, we added the following paragraphs in section “Results” and section “Discussions”.

Lines 238 to 254 in section “Results”

“Fig 4 shows the receiver operating characteristic (ROC) curves and the corresponding areas under the curves (AUCs) of the DT, SVM, and DNN models. Table 5 shows the detailed performance data of the models that delivered sensitivities at the 0.80 level and at the 0.90 level. It is observed that the DNN models marginally outperformed the DT models and the SVM models in terms of AUC. On the other hand, as shown in Table 5, if a high level of sensitivity is desirable, then the DT models significantly outperformed the DNN models and the SVM models in terms of the F1 score, which is the harmonic mean of the sensitivity (also called recall) and the positive predictive value (PPV, also called precision).

Based on the data shown in Table 5, it is conceivable that the DT model that delivered the sensitivity at the 0.90 level is the favorite choice due to two reasons. Firstly, the PPV with this particular DT model is significantly higher than the PPVs with the SVM model and the DNN model that delivered the same level of sensitivity. Therefore, in clinical applications, the number of false positive predicted by this DT model should be significantly lower than the numbers of false positive predicted by the SVM model and the DNN model with the same level of sensitivity. Secondly, the PPV with this DT model is almost the same as the PPV with the DT model that delivered the sensitivity at the 0.80 level. Accordingly, in the subsequent discussions, we will focus on the DT model that delivered the sensitivity at the 0.90 level.”

Line 270 to line 281 in section “Discussions”

“In this study, we have investigated how the frequencies of therapies can be exploited to build machine learning based prediction models for identifying children with development delay. Based on the experimental results observed, it is conceivable that the proposed approach can be widely exploited in clinical practices due to several reasons. Firstly, the performance observed with the prediction models developed in this study should meet the criteria acceptable by most physicians. For example, based on our experimental results, we can anticipate that the DT model shown in Table 5 can identify about 90.0% of the subjects who will suffer from DD in the future, while about 72% of the subjects predicted to be positive are actually true positives. Secondly, the features employed to build the prediction models can be obtained with essentially no costs. Therefore, the prediction models can be exploited to screen the subjects who may suffer from DD before advanced and costly diagnoses are carried out.”

Here are further comments to consider:

Comment 3: p.21, please provide more details about how the tree of four predictors were developed.
**Response:** In response to the comments provided by the reviewers, we have conducted comprehensive experiments to investigate the performance delivered by alternative types of prediction models. The experimental procedure is elaborated in section “**Development of prediction models and performance evaluation**” (lines 209 to 232). The new descriptions read: “In this study, we investigated the prediction performance of three categories of machine learning models, namely the DT models,^26-28^ the SVM models,^18^ and the DNN models^29^. The DT models are preferred by many clinicians due to the explicit decision rules output by the algorithm. On the other hand, the SVM models and the DNN models are two categories of the most advanced machine learning models that can generally outperform the DT models due to the non-linear transformations invoked in the prediction process. However, the non-linear transformations invoked also make it almost impossible for a user to comprehend how the prediction is made. As a result, many clinicians are reluctant to trust the models that work like a black box.

In order to obtain comprehensive pictures of how each category of prediction models performed, we employed alternative parameter settings to generate prediction models with different performance characteristics. Table 4 provides a summary of the software packages and alternative parameter settings employed to build the prediction models. Then, we conducted 10-fold cross validation to evaluate the performance characteristics of each prediction model generated. The performance metrics considered in this study include accuracy, sensitivity, specificity, positive predictive value (PPV as known as precision), and F1 score. The F1 score, which is the harmonic mean of the sensitivity and the PPV, is commonly employed in machine learning research and has increasingly been employed in biomedical research.^33^ Furthermore, for each category of prediction models, e.g., the DT models, the SVM models, or the DNN models, we evaluated its overall performance based on the area under the receiver operating characteristic (ROC) curve. In order to generate the ROC curve, we picked up the prediction model that delivered the highest F1 score at each level of sensitivity.”

Comment 4: p. 10, lines 61 …Developmental delay (DD) refers to a distinct set of early childhood developmental disabilities, and it is primarily diagnosed by assessing a child’s behavioral and mental capacities., reference is required
**Response:** Thank you for your suggestion. In the revised manuscript, reference 36 has been added to line 64.

Comment 5: P. 10, lines 84-87, references are required
**Response:** Thank you for your suggestion. In the revised manuscript, reference 37 has been added to line 85.

Comment 6: Line 180 'till2025/07/22', add space between **Response:** We apologize for the clerical error. In the revised manuscript, proper space has been added to line 171.

**Comments from Reviewer #2:**

Reviewer #2: This study developed an intelligent classification model using decision tree (DT), support vector machine (SVM), and deep neural network (DNN) techniques to identify children with developmental delay (DD) in Taiwan who require occupational therapy, with therapy service frequency features playing a crucial role. The study is interesting but can be improved in the following ways:
Comment 1: In lines 26-27, when discussing "various therapy services," it would be helpful to provide concrete examples (e.g., physical therapy, occupational therapy, or speech therapy). This will make it easier for readers to understand the scope of the services mentioned. **Response:** Thank you for your suggestion. In the revised manuscript, the three types of therapy services have been listed in lines 26-27 on page 2. The new sentence reads “In this study, we have investigated how the frequencies of three types of therapy, namely the physical therapy, the occupational therapy, and the speech therapy, received by a child can be exploited to predict whether the child suffers from DD or not.”

Comment 2: In Table 1, consider renaming the category "Accuracy" to "Performance." This adjustment would better encompass the variety of metrics provided by different detection methods, such as AUC and specificity, which are not strictly accuracy measures. **Response:** Thank you for your suggestion. In the revised manuscript, we have used “performance” to cover a variety of metrics, including Accuracy (AUC), sensitivity, specificity, positive predictive value (PPV), and F1 score. (Table 1. Lines 136-139)

Comment 3: In line 166, should "34862" refer to the number of visits rather than the number of patients? Clarifying this would avoid potential confusion for readers. In addition, are all those visits identified with DD? For example, the text states, “data of 34,862 patients with DD (mean age = 72.34 months) who visited the OPD of the study hospital.” Clarification on whether the visits or patients are associated with DD would enhance the accuracy of the description. **Response:** In the revised manuscript, the description in lines 166 to 168 has been revised to “The dataset used in this study comprised the medical records of the outpatients who visited the rehabilitation clinic of the study hospital with suspected DD between January 1, 2012, and December 31, 2016.” Furthermore, the description in lines 180 to 182 has been revised to “This study identified 2552 outpatients with age under 12 years who made one or more OPD visits. Among these patients, 1719 (67.4%) had DD. The total number of OPD visits was 34,862.”

Comment 4: Please ensure that in the tables, each word remains on a single line for better readability and presentation. **Response:** In the revised manuscript it is done. The words have been adjusted to fit a single line. We apologize for the confusing format.

Comment 5: Since feature selection was conducted, could you please provide a list of the features prior to selection? **Response:** Thank you for your suggestion. In the revised manuscript, we have added a section to describe the features that were employed. In lines 198 to 207 and Table 3 illustrate the feature selection process. The new sentence reads “In this study, we included 4 features in our dataset: gender and frequencies of OTS, PTS, and STS. In this respect, the frequency of a particular therapy service was defined to be the average number of services received by a patient in one year. Then, we conducted chi-squared tests to figure out whether a feature was correlated to the outcome variable ^23-25^. For feature gender, we carried out the chi-squared test of independence. For frequencies of OTS, PTS, and STS, we carried out the chi-squared test of goodness of fit with the null hypothesis set to the average frequency of patients with DD equal to the average frequency of patients without DD ^40^. Table 3 shows the p-values obtained. Accordingly, we included gender and frequencies of OTS, PTS, and STS to build the prediction models.”

Comment 6: Could you provide additional details, such as ROC curves, AUC values, or confusion matrices, to better illustrate the key results? **Response:** Thanks, you for your suggestion. In the revised manuscript, we have added a figure that shows the ROC curves of the 3 types of prediction models and provided the corresponding AUCs. Please refer to Fig 4. The ROC curves provide the users with overall pictures of how these different types of prediction modes performed with different parameter settings. Considering clinical applications, we speculate that physicians may be more interested in the prediction models that can deliver a high level of sensitivity. Therefore, in our revised manuscript, we added Table 5 on page 18, which shows the detailed performance data of the prediction models that deliver the sensitivity at the 0.80 and 0.90 levels and is attached here.

Comment 7: Since the frequency of therapy services is discussed in this paper, could you clarify what "frequency" refers to? For example, does it represent the number of services provided per year or over another period? Additionally, I am curious about the importance of the "Age" feature, as "frequency" might correlate with "Age." Could you conduct further experiments by removing the "Age" feature from the feature pool to assess its impact? This could provide valuable insights. **Response:** In the revised manuscript, we have added a section (lines 198 to 207). We believe that the descriptions provided in this section address the concerns. The section reads “In this study, we included 4 features in our dataset: gender and frequencies of OTS, PTS, and STS. In this respect, the frequency of a particular therapy service was defined to be the average number of services received by a patient in one year. Then, we conducted chi-squared tests to figure out whether a feature was correlated to the outcome variable. For feature gender, we carried out the chi-squared test of independence. For frequencies of OTS, PTS, and STS, we carried out the chi-squared test of goodness of fit with the null hypothesis set to the average frequency of patients with DD equal to the average frequency of patients without DD. Table 3 shows the p-values obtained. Accordingly, we included gender and frequencies of OTS, PTS, and STS to build the prediction models.”

For future studies, we have included the following discussions in section “Conclusions and Future Works” (lines 331 to 337), it is of interest to investigate the association between a child's age and the frequency of clinical therapy. It is of interest to investigate whether the therapy frequency peaks within a specific age range. The findings may support the principle of early intervention and carry significant clinical and therapeutic implications. Furthermore, identifying the age at which a child with DD is diagnosed, the specific clinical therapy received within rehabilitation, and the period of highest therapy concentration can facilitate our understanding of the most critical timeframe for early intervention.”

**Comments from Reviewer #3:**

Comment 1: This paper explores the use of machine learning models (decision trees, support vector machines, and deep neural networks) to classify pediatric developmental delay (DD) based on therapy service frequencies. Utilizing outpatient data from a Taiwanese hospital, the study identifies key predictors such as age, gender, and the frequencies of occupational therapy, physical therapy, and speech therapy services. The results show that the DT model provides high accuracy (72%), while the DNN model achieves high sensitivity (95%). The findings highlight the potential of machine learning in improving early detection and intervention strategies for children with DD. The manuscript provides a robust application of machine learning techniques to an important healthcare challenge, offering innovative insights into pediatric DD classification. Addressing the following points would enhance the study’s impact and clarity. **Response:** We sincerely appreciate the reviewer’s comments regarding our study.

Main Comments
Comment 2: Page 23, Tables 7 & 8: Provide details about the optimization process for the SVM-RBF model (e.g., grid search or cross-validation) to enhance reproducibility and clarify the rationale for parameter selection. **Response:** To prepare the revised manuscript, we conducted comprehensive experiments to obtain the ROC curves of the prediction models. Table 4, which is attached here, provides a comprehensive summary of the software packages and alternative parameter settings employed to build the DT models, the SVM models, and the DNN models.

**Table 4. The packages and parameters for DT, DNN, and SVM models**

| Model | Programming  Language | Package | Parameters |
| --- | --- | --- | --- |
| DT | Python | pandas  sklearn | split = "information",  prior = 0.1~0.9 with 0.008 step size,  cp = [0.005,0.004,0.003,0.002,0.001] |
| DNN | Python | TensorFlow | Input neurons = [4] for 4 features set,  Hidden neurons = [5,12,23]  Hidden layer = [2,4,8] |
| SVM | Python | sklearn  svm | Kernel = ['linear','rbf','poly'],  C = [0.01,0.1,1,3,10,15,20],  gamma='auto',  probability=True  Best for rbf kernal (cost = 10, gamma=0.25, epsilon=0.1) |

Comment 3: Lines 212–217, Discussion Section: Explain why the DNN model achieves high sensitivity (95%) but lower accuracy (70%) compared to the DT model. Discussing this trade-off would provide insight into the strengths and limitations of each model. **Response:** In the revised manuscript, we have added a figure that shows the ROC curves of the 3 types of prediction models and provided the corresponding AUCs. Please refer to Fig 4. The ROC curves provide the users with overall pictures of how these different types of prediction modes performed with different parameter settings. Considering clinical applications, we speculate that physicians may be more interested in the prediction models that can deliver a high level of sensitivity. Therefore, in our revised manuscript, we added Table 5 on page 18, which shows the detailed performance data of the prediction models that deliver the sensitivity at the 0.80 and 0.90 levels and is attached here.

Comment 4: Page 17, Table 2: Address the dataset’s significant gender imbalance (69.6% male) by discussing strategies to mitigate potential bias, such as weighted modeling or stratified sampling. **Response:** According to the literature, the percentage of male children who suffered from DD is higher than that of the female children. Since composition of our dataset conforms with the general observation, stratified sampling based on gender was not considered.

Comment 5: Page 25: Expand on how the findings might generalize to diverse populations and acknowledge limitations in broader applicability due to the retrospective nature of the data. **Response:** In section “Discussions”, we added the following paragraph (lines 298 to 305). “In summary, the major finding due to this study is that the frequencies of the therapies that a child has received provide valuable information for predicting whether the child suffers from DD. Due to the performance observed in the experiments and the fact that these features can be obtained essentially without any cost, it is conceivable that the prediction models built accordingly can be wide exploited in clinical practices and significantly improve the treatment outcomes of the children who suffer from DD. Though the study was based on a dataset collected in a hospital in Taiwan, we anticipate that the proposed method can be exploited to build accurate prediction models for populations in different countries with various race groups.”

Comment 6: Page 25, Discussion Section: Explore how therapy frequency as a predictor could be integrated into diagnostic frameworks or inform treatment plans, enhancing the paper's clinical relevance. **Response:** In the revised manuscript, we have added the following sentences to the first paragraph of section “Discussions” (lines 272 to 281). “Based on the experimental results observed, it is conceivable that the proposed approach can be widely exploited in clinical practices due to several reasons. Firstly, the performance observed with the prediction models developed in this study should meet the criteria acceptable by most physicians. For example, based on our experimental results, we can anticipate that the DT model shown in Table 5 can identify about 90.0% of the subjects who will suffer from DD in the future, while about 72% of the subjects predicted to be positive are actually true positives. Secondly, the features employed to build the prediction models can be obtained with essentially no costs. Therefore, the prediction models can be exploited to screen the subjects who may suffer from DD before advanced and costly diagnoses are carried out.”

Comment 7: Page 24: Provide a deeper exploration of ethical and practical considerations, such as data privacy, prediction transparency, and integration into clinical workflows. **Response:** In the revised manuscript, we have the following descriptions regarding data privacy (lines 166 to 171): “The dataset used in this study comprised the medical records of the outpatients who visited the rehabilitation clinic of the study hospital with suspected DD between January 1, 2012, and December 31, 2016.The Institutional Review Board of the En Chu Kong Hospital has reviewed the above documents and approved the study on 2024/07/23 (ECK-IRB Number: ECKIRB1130501). This approval is valid till 2025/07/22.” Furthermore, we have added the following sentences to the first paragraph of section “Discussions” (lines 272 to 281): “Based on the experimental results observed, it is conceivable that the proposed approach can be widely exploited in clinical practices due to several reasons. Firstly, the performance observed with the prediction models developed in this study should meet the criteria acceptable by most physicians. For example, based on our experimental results, we can anticipate that the DT model shown in Table 5 can identify about 90.0% of the subjects who will suffer from DD in the future, while about 72% of the subjects predicted to be positive are actually true positives. Secondly, the features employed to build the prediction models can be obtained with essentially no costs. Therefore, the prediction models can be exploited to screen the subjects who may suffer from DD before advanced and costly diagnoses are carried out.”

Minor Comments
Comment 8: Figure 1, Page 33, Lines 181–186: Add a clearer caption detailing the inclusion and exclusion criteria for the flow diagram to improve understanding of the study design. **Response:** Thank you for your suggestion. A clearer caption has been added in Figure 1. [“Figures caption”, Pages 27-28, Lines 457–462]

Comment 9: Figure 3, Page 35: Include a description of the significance of each decision tree split (e.g., OT vs. PT frequencies) in both the figure caption and the main text. **Response:** Thank you for your suggestion. The figure caption and main text have been added to Fig 3.

Comment 10: Page 24: Expand the discussion of future research to include advanced techniques like alternative DNN architectures or multimodal data (e.g., imaging and behavioral assessments). **Response:** In the revised manuscript, we have included the following discussion (lines 322 to 326) “As this study has revealed that the frequencies of the therapies that a child has received provide valuable information for predicting whether the child suffers from DD, it is conceivable that we can built more accurate prediction models by integrating these features with other clinical assessment scores and the records of advanced medical examinations such as brain imaging, electroencephalograms, and etc.”

Comment 11: Page 18, Lines 200–207: Improve transitions between steps in the methodology section (e.g., from feature selection to model training) for better clarity and readability. **Response:** We have revised the descriptions about feature selection (lines 199 to 207). The new paragraph reads “In this study, we included 4 features in our dataset: gender and frequencies of OTS, PTS, and STS. In this respect, the frequency of a particular therapy service was defined to be the average number of services received by a patient in one year. Then, we conducted chi-squared tests to figure out whether a feature was correlated to the outcome variable ^23-25^. For feature gender, we carried out the chi-squared test of independence. For frequencies of OTS, PTS, and STS, we carried out the chi-squared test of goodness of fit with the null hypothesis set to the average frequency of patients with DD equal to the average frequency of patients without DD ^40^. Table 3 shows the p-values obtained. Accordingly, we included gender and frequencies of OTS, PTS, and STS to build the prediction models.”. Furthermore, we have revised section “Development of prediction models and performance evaluation” (lines 209 to 235). The new paragraph reads “In this study, we investigated the prediction performance of three categories of machine learning models, namely the DT models,^26-28^ the SVM models,^18^ and the DNN models and the DNN models ^29^. The DT models are preferred by many clinicians due to the explicit decision rules output by the algorithm. On the other hand, the SVM models and the DNN models are two categories of the most advanced machine learning models that can generally outperform the DT models due to the non-linear transformations invoked in the prediction process. However, the non-linear transformations invoked also make it almost impossible for a user to comprehend how the prediction is made. As a result, many clinicians are reluctant to trust the models that work like a black box.

In order to obtain comprehensive pictures of how each category of prediction models performed, we employed alternative parameter settings to generate prediction models with different performance characteristics. Table 4 provides a summary of the software packages and alternative parameter settings employed to build the prediction models. Then, we conducted 10-fold cross validation to evaluate the performance characteristics of each prediction model generated. The performance metrics considered in this study include accuracy, sensitivity, specificity, positive predictive value (PPV as known as precision), and F1 score. The F1 score, which is the harmonic mean of the sensitivity and the PPV, is commonly employed in machine learning research and has increasingly been employed in biomedical research.^33^ Furthermore, for each category of prediction models, e.g., the DT models, the SVM models, or the DNN models, we evaluated its overall performance based on the area under the receiver operating characteristic (ROC) curve. In order to generate the ROC curve, we picked up the prediction model that delivered the highest F1 score at each level of sensitivity.”

Comment 12: Page 25: Elaborate on the potential impact of missing data or unmeasured confounding variables in the limitations section. **Response:** We have included the following discussion in the limitations section. (Lines 314 to 317). “Fourthly, due to the strict flowchart employed by the hospital, the outpatient medical records from which our dataset was derived are highly accurate and include minimal missing data and few unmeasured confounding variables.”

Comment 13: Encourage the authors to make their machine learning models publicly available, even without the dataset, to enhance reproducibility and allow others to validate and build upon their work. This aligns with open science principles and supports further advancements in the field. **Response:** We will be pleased to make the prediction models publicly available for validation. We sincerely appreciate the reviewers' recognition of our work.
