# Supplementary material for: Detection of pediatric developmental delay with machine learning technologies
Source: PLoS One. 2025 May 20;20(5):e0324204. doi: 10.1371/journal.pone.0324204 (PMC12091767; doi:10.1371/journal.pone.0324204)
Supplement: Attachment — Submitted filename: Response to Reviewers-20250416.docx [file pone.0324204.s005.docx]

**Comment 1 from reviewer 1:**

The authors clearly have carefully considered the comments provided, and took reasonable attempts to address the comments; which resulted a substantial improvement of this version of manuscript.

Particularly:
The inclusion of figure 4, table 5, and additional text describing the results on p.17-18, have provided valuable information for the readers in terms of how well the proposed three models in predicting DD. The authors have subsequently discussed the results and its implication in the discussion section, as well as drawing conclusion based on the current findings. Thus, this revised manuscript has provide valuable insight into this academic disciplines.

**Response:**

Thank you very much for your positive comments.

**[ Major concern ]**

**Comment 2 from reviewer 1:**

I appreciate the new added text is to respond to Reviewer#2 1st comment, to provide some examples of therapy. However, currently it may create a confusion for the readers.
In objective: it mentioned 'we have investigated how the frequencies of three types of therapy, namely the physical therapy, the occupational therapy, and the speech therapy, received by a child can be exploited to predict whether the child suffers from DD or not'. And then the next sentence is '…The effectiveness of the proposed approach…'
These two sentences seem to imply the study aimed to investigate and compare the frequencies of three types of therapy predict DD. But in the method, results and conclusion sections, it mainly discussed different machine learning models.

Reading the manuscript, should the objective of the study is about the effectiveness of different machine learning based prediction models that predict DD in clinical settings? If it is, it seems that it is not reflected clearly in the objective section.

**Response:**

In this study, we have aimed to investigated how the frequencies of three types of therapy, namely the physical therapy, the occupational therapy, and the speech therapy, received by a child can be exploited to predict whether the child suffers from DD or not. The reason why we built alternative categories of prediction models was to investigate how the DT models performed in our applications in comparison with advanced machine learning models such as the SVM models and the DNN models. As the users of DT models can easily interpret how the predictions are made by examining the explicit decision rules output by the algorithm, the DT models are favorite for applications in which the DT models can deliver the same level of performance as advanced machine learning models. In order to clarify our objective, we have added the following statements to the first paragraph of section “Development of prediction models and performance evaluation” (lines 229-234): Therefore, it is of interest to investigate how the performance of alternative categories of machine learning models compares. If the performance of the DT models observed in the experiments is comparable with the performance of the advanced machine learning models, which was observed in our recent studies [43-44], then the DT models are favorite due to explicit decision rules output by the algorithm.

Furthermore, we have added the following statement to the second paragraph of section “Discussion” (lines 306-307):

Fortunately, for our applications, we do not need to trade performance for the interpretability of the prediction model.

**Comment 3 from reviewer 1:**

In the revised text, limitations section:
Line 315: 'Fourthly, due to the strict flowchart employed by the hospital, the outpatient medical records from which our dataset was derived are highly accurate and include minimal missing data and few unmeasured confounding variables.'
I found hard to understand why this is a limitation by reading the manuscript, especially in terms of 'highly accurate and minimal missing data'. If 'few unmeasured confounding variables' are the limitation, it would be great to have some elaboration, what they are (or could be), and how it may be a limitation. If 'highly accurate and minimal missing data' is actually a strength, suggest the author could explicitly acknowledging that it's the strength, or need not to mention.

**Response:**

We apologize for the confusion. We have deleted this statement from the first paragraph of section “Discussion”. Instead, we added the following statement to the second paragraph of section “Data collection and outcome measurement

”(lines 191-193):

It is observed that due to the strict flowchart employed by the hospital, the outpatient medical records from which our dataset was derived are highly accurate and include minimal missing data and few unmeasured confounding variables.

**[ Minor concern ]**

**Comment 4 from reviewer 1:**
Table 1, suggest improve consistency and readability of how data is reported, e.g., in number of samples:
Row 1: 'Total 876', row 2 '292 samples', later on '47 subjects', suggest to have more consistency; also some row reported overall sample size, and then break into different sub-group 292 Samples = 141 ASD+151
Non-ASD (4-11Yrs) , some only report sub-groups directly without overall N, '22 ASD Children, 16 Normal Children'
Suggest there should be abbreviations of all stat terms used in the table, e.g., NMI, DMN, AUC, etc.
**Response:**

Thank you for your suggestions. We have added abbreviations for all statistical terms used in the table and revised the description of sample sizes to enhance data clarity and readability. (lines 136-143)

**Comment 5 from reviewer 1:**

Methods
Line 155, '…were previously given a diagnosis based on the criteria established in the…'
Can the author specify what diagnosis, e.g., DD? Or any diagnosis within DSM-IV TR, or developmental related Dx?
**Response:**

Thank you for your feedback. Accordingly, we have included the following statement: “For example, the DSM-5-TR defines autism spectrum disorder (ASD) as involving persistent deficits in social communication across multiple environments, as outlined in the relevant diagnostic criterion” (lines 162–164)

**Comment 6 from reviewer 1:**
The use of the phrase 'suffer from DD' throughout the manuscript, while it may be valid; taking from a neuro-diversity affirming lens, would the authors open to consider using a more neutral word, e.g., 'will have DD, or will be diagnosed with DD'?

**Response:**

Thank you for your suggestions. We have replaced the phrase 'suffer from DD' with 'will/may develop DD' throughout the manuscript to adopt more neutral and objective language.
