# Supplementary material for: Detection of pediatric developmental delay with machine learning technologies
Source: PLoS One. 2025 May 20;20(5):e0324204. doi: 10.1371/journal.pone.0324204 (PMC12091767; doi:10.1371/journal.pone.0324204)
Supplement: Peer review history

# Author comment: Author response to Decision Letter 0

**DOI:** 10.1371/journal.pone.0324204.r001

28 Aug 2024

# Peer review: Decision Letter 0

**DOI:** 10.1371/journal.pone.0324204.r002

**Authors:** Zeheng Wang

2 Jan 2025

PONE-D-24-35849Detection of Pediatric Developmental Delay with Machine Learning TechnologiesPLOS ONE

Dear Dr. Oyang,

Thank you for submitting your manuscript to PLOS ONE. After careful consideration, we feel that it has merit but does not fully meet PLOS ONE’s publication criteria as it currently stands. Therefore, we invite you to submit a revised version of the manuscript that addresses the points raised during the review process.

Please submit your revised manuscript by Feb 16 2025 11:59PM. If you will need more time than this to complete your revisions, please reply to this message or contact the journal office at plosone@plos.org . When you're ready to submit your revision, log on to https://www.editorialmanager.com/pone/ and select the 'Submissions Needing Revision' folder to locate your manuscript file.

Please include the following items when submitting your revised manuscript:

- A rebuttal letter that responds to each point raised by the academic editor and reviewer(s). You should upload this letter as a separate file labeled 'Response to Reviewers'.
- A marked-up copy of your manuscript that highlights changes made to the original version. You should upload this as a separate file labeled 'Revised Manuscript with Track Changes'.
- An unmarked version of your revised paper without tracked changes. You should upload this as a separate file labeled 'Manuscript'.

If you would like to make changes to your financial disclosure, please include your updated statement in your cover letter. Guidelines for resubmitting your figure files are available below the reviewer comments at the end of this letter.

If applicable, we recommend that you deposit your laboratory protocols in protocols.io to enhance the reproducibility of your results. Protocols.io assigns your protocol its own identifier (DOI) so that it can be cited independently in the future. For instructions see: https://journals.plos.org/plosone/s/submission-guidelines#loc-laboratory-protocols . Additionally, PLOS ONE offers an option for publishing peer-reviewed Lab Protocol articles, which describe protocols hosted on protocols.io. Read more information on sharing protocols at https://plos.org/protocols?utm_medium=editorial-email&utm_source=authorletters&utm_campaign=protocols .

We look forward to receiving your revised manuscript.

Kind regards,

Zeheng Wang

Academic Editor

PLOS ONE

Journal Requirements:

When submitting your revision, we need you to address these additional requirements.

1. Please ensure that your manuscript meets PLOS ONE's style requirements, including those for file naming. The PLOS ONE style templates can be found at 

https://journals.plos.org/plosone/s/file?id=wjVg/PLOSOne_formatting_sample_main_body.pdf and 

https://journals.plos.org/plosone/s/file?id=ba62/PLOSOne_formatting_sample_title_authors_affiliations.pdf

2. Please note that PLOS ONE has specific guidelines on code sharing for submissions in which author-generated code underpins the findings in the manuscript. In these cases, we expect all author-generated code to be made available without restrictions upon publication of the work. Please review our guidelines at https://journals.plos.org/plosone/s/materials-and-software-sharing#loc-sharing-code and ensure that your code is shared in a way that follows best practice and facilitates reproducibility and reuse.

3. Please provide a complete Data Availability Statement in the submission form, ensuring you include all necessary access information or a reason for why you are unable to make your data freely accessible. If your research concerns only data provided within your submission, please write "All data are in the manuscript and/or supporting information files" as your Data Availability Statement.

4. PLOS requires an ORCID iD for the corresponding author in Editorial Manager on papers submitted after December 6th, 2016. Please ensure that you have an ORCID iD and that it is validated in Editorial Manager. To do this, go to ‘Update my Information’ (in the upper left-hand corner of the main menu), and click on the Fetch/Validate link next to the ORCID field. This will take you to the ORCID site and allow you to create a new iD or authenticate a pre-existing iD in Editorial Manager.

5. Please include captions for your Supporting Information files at the end of your manuscript, and update any in-text citations to match accordingly. Please see our Supporting Information guidelines for more information: http://journals.plos.org/plosone/s/supporting-information. 

[Note: HTML markup is below. Please do not edit.]

Reviewers' comments:

Reviewer's Responses to Questions

**Comments to the Author**

1. Is the manuscript technically sound, and do the data support the conclusions?

The manuscript must describe a technically sound piece of scientific research with data that supports the conclusions. Experiments must have been conducted rigorously, with appropriate controls, replication, and sample sizes. The conclusions must be drawn appropriately based on the data presented. 

Reviewer #1: Partly

Reviewer #2: Yes

Reviewer #3: Yes

**********

2. Has the statistical analysis been performed appropriately and rigorously? 

Reviewer #1: I Don't Know

Reviewer #2: Yes

Reviewer #3: Yes

**********

3. Have the authors made all data underlying the findings in their manuscript fully available?

The PLOS Data policy requires authors to make all data underlying the findings described in their manuscript fully available without restriction, with rare exception (please refer to the Data Availability Statement in the manuscript PDF file). The data should be provided as part of the manuscript or its supporting information, or deposited to a public repository. For example, in addition to summary statistics, the data points behind means, medians and variance measures should be available. If there are restrictions on publicly sharing data—e.g. participant privacy or use of data from a third party—those must be specified.

Reviewer #1: Yes

Reviewer #2: Yes

Reviewer #3: Yes

**********

4. Is the manuscript presented in an intelligible fashion and written in standard English?

PLOS ONE does not copyedit accepted manuscripts, so the language in submitted articles must be clear, correct, and unambiguous. Any typographical or grammatical errors should be corrected at revision, so please note any specific errors here.

Reviewer #1: Yes

Reviewer #2: No

Reviewer #3: Yes

**********

5. Review Comments to the Author

Please use the space provided to explain your answers to the questions above. You may also include additional comments for the author, including concerns about dual publication, research ethics, or publication ethics. (Please upload your review as an attachment if it exceeds 20,000 characters)

Reviewer #1: Thanks for the opportunity to review the current study, which described using machine learning technologies to identify patients with developmental delay. This is an important area that is worthwhile for further research. The study has a particular strength in using a large sample size in real-life settings for the model development.

The study is generally well-written. It covered key concepts in in the introduction. It has also provided a comprehensive summary of previous literatures.

However, there is two major concerns that affect the paper's contribution in its current state.

1. In the discussion section, there is a need to discuss the results in more details, e.g., the use of different models (SVM, RBF, DNN, etc.). Currently, there are lots of important aspects of the findings have not been well discussed. Therefore, it makes the limitation, future research directions, and conclusion not strongly connected and supported by the current findings.

2. Whereas the authors mentioned sensitivity and accuracy in the results section, the authors have more focus on reporting and discussing sensitivity. It could be important to discuss specificity in more details in the both results and discussion section. Specificity is also an key factors determining overall accuracy. Particularly, in the results section, the authors reported, 'DT model achieved a higher accuracy of up to 72%', 'DNN model achieved a higher sensitivity of up to 95% (accuracy = 0.70, F1 score = 0.81).', 'RBF kernel can identify problems with an accuracy of up to 71% (sensitivity = 0.93, F1 score = 0.81)' However, RBF actually has a very low specificity of 0.233. DT maintains a relatively higher specificity of o.61, and DNN has a very low specificity of 0.19. These information are also very important in determining the accuracy of the model and suggest to be further considered and discussed further.

Here are further comments to consider:

p.21, please provide more details about how the tree of four predictors were developed.

p. 10 , lines 61 …Developmental delay (DD) refers to a distinct set of early childhood developmental disabilities, and it is primarily diagnosed by assessing a child’s behavioral and mental capacities., reference is required

P. 10, lines 84-87, references are required

Line 170 'till2025/07/22', add space between

Reviewer #2: This study developed an intelligent classification model using decision tree (DT), support vector machine (SVM), and deep neural network (DNN) techniques to identify children with developmental delay (DD) in Taiwan who require occupational therapy, with therapy service frequency features playing a crucial role. The study is interesting but can be improved in the following ways:

1. In line 31, when discussing "various therapy services," it would be helpful to provide concrete examples (e.g., physical therapy, occupational therapy, or speech therapy). This will make it easier for readers to understand the scope of the services mentioned.

2. In Table 1, consider renaming the category "Accuracy" to "Performance." This adjustment would better encompass the variety of metrics provided by different detection methods, such as AUC and specificity, which are not strictly accuracy measures.

3. In line 166, should "34862" refer to the number of visits rather than the number of patients? Clarifying this would avoid potential confusion for readers.

In addition, are all those visits identified with DD? For example, the text states, “data of 34,862 patients with DD (mean age = 72.34 months) who visited the OPD of the study hospital.” Clarification on whether the visits or patients are associated with DD would enhance the accuracy of the description.

4. Please ensure that in the tables, each word remains on a single line for better readability and presentation.

5. Since feature selection was conducted, could you please provide a list of the features prior to selection?

6. Could you provide additional details, such as ROC curves, AUC values, or confusion matrices, to better illustrate the key results?

7. Since the frequency of therapy services is discussed in this paper, could you clarify what "frequency" refers to? For example, does it represent the number of services provided per year or over another period? Additionally, I am curious about the importance of the "Age" feature, as "frequency" might correlate with "Age." Could you conduct further experiments by removing the "Age" feature from the feature pool to assess its impact? This could provide valuable insights.

Reviewer #3: This paper explores the use of machine learning models (decision trees, support vector machines, and deep neural networks) to classify pediatric developmental delay (DD) based on therapy service frequencies. Utilizing outpatient data from a Taiwanese hospital, the study identifies key predictors such as age, gender, and the frequencies of occupational therapy, physical therapy, and speech therapy services. The results show that the DT model provides high accuracy (72%), while the DNN model achieves high sensitivity (95%). The findings highlight the potential of machine learning in improving early detection and intervention strategies for children with DD. The manuscript provides a robust application of machine learning techniques to an important healthcare challenge, offering innovative insights into pediatric DD classification. Addressing the following points would enhance the study’s impact and clarity.

Main Comments

Page 23, Tables 7 & 8: Provide details about the optimization process for the SVM-RBF model (e.g., grid search or cross-validation) to enhance reproducibility and clarify the rationale for parameter selection.

Lines 212–217, Discussion Section: Explain why the DNN model achieves high sensitivity (95%) but lower accuracy (70%) compared to the DT model. Discussing this trade-off would provide insight into the strengths and limitations of each model.

Page 17, Table 2: Address the dataset’s significant gender imbalance (69.6% male) by discussing strategies to mitigate potential bias, such as weighted modeling or stratified sampling.

Page 25: Expand on how the findings might generalize to diverse populations and acknowledge limitations in broader applicability due to the retrospective nature of the data.

Page 25, Discussion Section: Explore how therapy frequency as a predictor could be integrated into diagnostic frameworks or inform treatment plans, enhancing the paper's clinical relevance.

Page 24: Provide a deeper exploration of ethical and practical considerations, such as data privacy, prediction transparency, and integration into clinical workflows.

Minor Comments

Figure 1, Page 33, Lines 181–186: Add a clearer caption detailing the inclusion and exclusion criteria for the flow diagram to improve understanding of the study design.

Figure 3, Page 35: Include a description of the significance of each decision tree split (e.g., OT vs. PT frequencies) in both the figure caption and the main text.

Page 24: Expand the discussion of future research to include advanced techniques like alternative DNN architectures or multimodal data (e.g., imaging and behavioral assessments).

Page 18, Lines 200–207: Improve transitions between steps in the methodology section (e.g., from feature selection to model training) for better clarity and readability.

Page 25: Elaborate on the potential impact of missing data or unmeasured confounding variables in the limitations section.

Encourage the authors to make their machine learning models publicly available, even without the dataset, to enhance reproducibility and allow others to validate and build upon their work. This aligns with open science principles and supports further advancements in the field.

**********

6. PLOS authors have the option to publish the peer review history of their article (what does this mean? ). If published, this will include your full peer review and any attached files.

If you choose “no”, your identity will remain anonymous but your review may still be made public.

**Do you want your identity to be public for this peer review?** For information about this choice, including consent withdrawal, please see our Privacy Policy .

Reviewer #1: No

Reviewer #2: No

Reviewer #3: No

**********

[NOTE: If reviewer comments were submitted as an attachment file, they will be attached to this email and accessible via the submission site. Please log into your account, locate the manuscript record, and check for the action link "View Attachments". If this link does not appear, there are no attachment files.]

While revising your submission, please upload your figure files to the Preflight Analysis and Conversion Engine (PACE) digital diagnostic tool, https://pacev2.apexcovantage.com/ . PACE helps ensure that figures meet PLOS requirements. To use PACE, you must first register as a user. Registration is free. Then, login and navigate to the UPLOAD tab, where you will find detailed instructions on how to use the tool. If you encounter any issues or have any questions when using PACE, please email PLOS at figures@plos.org . Please note that Supporting Information files do not need this step.

# Author comment: Author response to Decision Letter 0

**DOI:** 10.1371/journal.pone.0324204.r003

20 Mar 2025

Comment 1 from Reviewer #1:

Thanks for the opportunity to review the current study, which described using machine learning technologies to identify patients with developmental delay. This is an important area that is worthwhile for further research. The study has a particular strength in using a large sample size in real-life settings for the model development.

The study is generally well-written. It covered key concepts in in the introduction. It has also provided a comprehensive summary of previous literatures.

However, there is two major concerns that affect the paper's contribution in its current state.

Comment 1: In the discussion section, there is a need to discuss the results in more details, e.g., the use of different models (SVM, RBF, DNN, etc.). Currently, there are lots of important aspects of the findings have not been well discussed. Therefore, it makes the limitation, future research directions, and conclusion not strongly connected and supported by the current findings.

Comment 2: Whereas the authors mentioned sensitivity and accuracy in the results section, the authors have more focus on reporting and discussing sensitivity. It could be important to discuss specificity in more details in the both results and discussion section. Specificity is also an key factors determining overall accuracy. Particularly, in the results section, the authors reported, 'DT model achieved a higher accuracy of up to 72%', 'DNN model achieved a higher sensitivity of up to 95% (accuracy = 0.70, F1 score = 0.81).', 'RBF kernel can identify problems with an accuracy of up to 71% (sensitivity = 0.93, F1 score = 0.81)' However, RBF actually has a very low specificity of 0.233. DT maintains a relatively higher specificity of o.61, and DNN has a very low specificity of 0.19. These information are also very important in determining the accuracy of the model and suggest to be further considered and discussed further.

Responses:

1. We have revised the "4" section significantly. Please refer to the part from line 270 on page 18 to line 297 on page 19. We have also extracted this part and attached it here.

"In this study, we have investigated how the frequencies of therapies can be exploited to build machine learning based prediction models for identifying children with development delay. Based on the experimental results observed, it is conceivable that the proposed approach can be widely exploited in clinical practices due to several reasons. Firstly, the performance observed with the prediction models developed in this study should meet the criteria acceptable by most physicians. For example, based on our experimental results, we can anticipate that the DT model shown in Table 5 can identify about 90.0% of the subjects who will suffer from DD in the future, while about 72% of the subjects predicted to be positive are true positives. Secondly, the features employed to build the prediction models can be obtained with essentially no costs. Therefore, the prediction models can be exploited to screen the subjects who may suffer from DD before advanced and costly diagnoses are carried out.

The experimental results also demonstrate that for the applications targeted by this study we do not need to trade performance for the interpretability of the prediction model. The F1 scores presented in Table 5 show that the DT models that delivered the sensitivity at the 0.90 level and at the 0.80 level outperformed the DNN models and the SVM model that delivered the sensitivity at the same level. For most applications, it is typical that advanced machine learning based prediction models such as the DNN models and the SVM models outperform the DT models due to the non-linear transformations invoked. However, the non-linear transformations invoked also make it almost impossible for a user to figure out how the prediction is made.

The DT structure shown in Fig3 illustrates how a user can examine the structure to figure out the decision rules followed by the prediction model to make predictions. Furthermore, the ratio of between the number of positive subjects and the number of negative subjects at each leaf node specifies how likely a subject that meets the criteria corresponding to the path to this particular leaf node suffers from DD. For example, the probability for the subject illustrated in Fig 3 to suffer from DD is 0.727. In clinical practice, a physician can refer to this specific probability and his/her clinical experiences to make the final diagnosis."

2. In the revised manuscript, we have added a figure that shows the ROC curves of the 3 types of prediction models and provided the corresponding AUCs. Please refer to Fig 4. The ROC curves provide the users with overall pictures of how these different types of prediction modes performed with different parameter settings. Considering clinical applications, we speculate that physicians may be more interested in the prediction models that can deliver a high level of sensitivity. Therefore, in our revised manuscript, we added Table 5 on page 18, which shows the detailed performance data of the prediction models that deliver the sensitivity at the 0.80 and 0.90 levels and is attached here. Furthermore, we added the following paragraphs in section "Results" and section "Discussions".

Lines 238 to 254 in section "Results"

"Fig 4 shows the receiver operating characteristic (ROC) curves and the corresponding areas under the curves (AUCs) of the DT, SVM, and DNN models. Table 5 shows the detailed performance data of the models that delivered sensitivities at the 0.80 level and at the 0.90 level. It is observed that the DNN models marginally outperformed the DT models and the SVM models in terms of AUC. On the other hand, as shown in Table 5, if a high level of sensitivity is desirable, then the DT models significantly outperformed the DNN models and the SVM models in terms of the F1 score, which is the harmonic mean of the sensitivity (also called recall) and the positive predictive value (PPV, also called precision).

Based on the data shown in Table 5, it is conceivable that the DT model that delivered the sensitivity at the 0.90 level is the favorite choice due to two reasons. Firstly, the PPV with this particular DT model is significantly higher than the PPVs with the SVM model and the DNN model that delivered the same level of sensitivity. Therefore, in clinical applications, the number of false positive predicted by this DT model should be significantly lower than the numbers of false positive predicted by the SVM model and the DNN model with the same level of sensitivity. Secondly, the PPV with this DT model is almost the same as the PPV with the DT model that delivered the sensitivity at the 0.80 level. Accordingly, in the subsequent discussions, we will focus on the DT model that delivered the sensitivity at the 0.90 level."

Line 270 to line 281 in section "Discussions"

"In this study, we have investigated how the frequencies of therapies can be exploited to build machine learning based prediction models for identifying children with development delay. Based on the experimental results observed, it is conceivable that the proposed approach can be widely exploited in clinical practices due to several reasons. Firstly, the performance observed with the prediction models developed in this study should meet the criteria acceptable by most physicians. For example, based on our experimental results, we can anticipate that the DT model shown in Table 5 can identify about 90.0% of the subjects who will suffer from DD in the future, while about 72% of the subjects predicted to be positive are actually true positives. Secondly, the features employed to build the prediction models can be obtained with essentially no costs. Therefore, the prediction models can be exploited to screen the subjects who may suffer from DD before advanced and costly diagnoses are carried out."

Here are further comments to consider:

Comment 3: p.21, please provide more details about how the tree of four predictors were developed.

Response: In response to the comments provided by the reviewers, we have conducted comprehensive experiments to investigate the performance delivered by alternative types of prediction models. The experimental procedure is elaborated in section "Development of prediction models and performance evaluation" (lines 209 to 232). The new descriptions read: "In this study, we investigated the prediction performance of three categories of machine learning models, namely the DT models, the SVM models, and the DNN models. The DT models are preferred by many clinicians due to the explicit decision rules output by the algorithm. On the other hand, the SVM models and the DNN models are two categories of the most advanced machine learning models that can generally outperform the DT models due to the non-linear transformations invoked in the prediction process. However, the non-linear transformations invoked also make it almost impossible for a user to comprehend how the prediction is made. As a result, many clinicians are reluctant to trust the models that work like a black box.

In order to obtain comprehensive pictures of how each category of prediction models performed, we employed alternative parameter settings to generate prediction models with different performance characteristics. Table 4 provides a summary of the software packages and alternative parameter settings employed to build the prediction models. Then, we conducted 10-fold cross validation to evaluate the performance characteristics of each prediction model generated. The performance metrics considered in this study include accuracy, sensitivity, specificity, positive predictive value (PPV as known as precision), and F1 score. The F1 score, which is the harmonic mean of the sensitivity and the PPV, is commonly employed in machine learning research and has increasingly been employed in biomedical research. Furthermore, for each category of prediction models, e.g., the DT models, the SVM models, or the DNN models, we evaluated its overall performance based on the area under the receiver operating characteristic (ROC) curve. In order to generate the ROC curve, we picked up the prediction model that delivered the highest F1 score at each level of sensitivity."

Comment 4: p. 10, lines 61 …Developmental delay (DD) refers to a distinct set of early childhood developmental disabilities, and it is primarily diagnosed by assessing a child’s behavioral and mental capacities., reference is required

Response: Thank you for your suggestion. In the revised manuscript, reference 36 has been added to line 64.

Comment 5: P. 10, lines 84-87, references are required

Response: Thank you for your suggestion. In the revised manuscript, reference 37 has been added to line 85.

Comment 6: Line 180 'till2025/07/22', add space between

Response: We apologize for the clerical error. In the revised manuscript, proper space has been added to line 171.

Comments from Reviewer #2:

Reviewer #2: This study developed an intelligent classification model using decision tree (DT), support vector machine (SVM), and deep neural network (DNN) techniques to identify children with developmental delay (DD) in Taiwan who require occupational therapy, with therapy service frequency features playing a crucial role. The study is interesting but can be improved in the following ways:

Comment 1: In lines 26-27, when discussing "various therapy services," it would be helpful to provide concrete examples (e.g., physical therapy, occupational therapy, or speech therapy). This will make it easier for readers to understand the scope of the services mentioned. Response: Thank you for your suggestion. In the revised manuscript, the three types of therapy services have been listed in lines 26-27 on page 2. The new sentence reads "In this study, we have investigated how the frequencies of three types of therapy, namely the physical therapy, the occupational therapy, and the speech therapy, received by a child can be exploited to predict whether the child suffers from DD or not."

Comment 2: In Table 1, consider renaming the category "Accuracy" to "Performance." This adjustment would better encompass the variety of metrics provided by different detection methods, such as AUC and specificity, which are not strictly accuracy measures. Response: Thank you for your suggestion. In the revised manuscript, we have used "performance" to cover a variety of metrics, including Accuracy (AUC), sensitivity, specificity, positive predictive value (PPV), and F1 score. (Table 1. Lines 136-139)

Comment 3: In line 166, should "34862" refer to the number of visits rather than the number of patients? Clarifying this would avoid potential confusion for readers. In addition, are all those visits identified with DD? For example, the text states, “data of 34,862 patients with DD (mean age = 72.34 months) who visited the OPD of the study hospital.” Clarification on whether the visits or patients are associated with DD would enhance the accuracy of the description.

Response: In the revised manuscript, the description in lines 166 to 168 has been revised to "The dataset used in this study comprised the medical records of the outpatients who visited the rehabilitation clinic of the study hospital with suspected DD between January 1, 2012, and December 31, 2016." Furthermore, the description in lines 180 to 182 has been revised to "This study identified 2552 outpatients with age under 12 years who made one or more OPD visits. Among these patients, 1719 (67.4%) had DD. The total number of OPD visits was 34,862."

Comment 4: Please ensure that in the tables, each word remains on a single line for better readability and presentation. Response: In the revised manuscript it is done. The words have been adjusted to fit a single line. We apologize for the confusing format.

Comment 5: Since feature selection was conducted, could you please provide a list of the features prior to selection? Response: Thank you for your suggestion. In the revised manuscript, we have added a section to describe the features that were employed. In lines 198 to 207 and Table 3 illustrate the feature selection process. The new sentence reads "In this study, we included 4 features in our dataset: gender and frequencies of OTS, PTS, and STS. In this respect, the frequency of a particular therapy service was defined to be the average number of services received by a patient in one year. Then, we conducted chi-squared tests to figure out whether a feature was correlated to the outcome variable. For feature gender, we carried out the chi-squared test of independence. For frequencies of OTS, PTS, and STS, we carried out the chi-squared test of goodness of fit with the null hypothesis set to the average frequency of patients with DD equal to the average frequency of patients without DD. Table 3 shows the p-values obtained. Accordingly, we included gender and frequencies of OTS, PTS, and STS to build the prediction models."

Comment 6: Could you provide additional details, such as ROC curves, AUC values, or confusion matrices, to better illustrate the key results? Response: Thanks, you for your suggestion. In the revised manuscript, we have added a figure that shows the ROC curves of the 3 types of prediction models and provided the corresponding AUCs. Please refer to Fig 4. The ROC curves provide the users with overall pictures of how these different types of prediction modes performed with different parameter settings. Considering clinical applications, we speculate that physicians may be more interested

Attachment Submitted filename: Response to Reviewers.docx

# Peer review: Decision Letter 1

**DOI:** 10.1371/journal.pone.0324204.r004

**Authors:** Zeheng Wang

4 Apr 2025

PONE-D-24-35849R1Detection of Pediatric Developmental Delay with Machine Learning TechnologiesPLOS ONE

Dear Dr. Oyang,

Thank you for submitting your manuscript to PLOS ONE. After careful consideration, we feel that it has merit but does not fully meet PLOS ONE’s publication criteria as it currently stands. Therefore, we invite you to submit a revised version of the manuscript that addresses the points raised during the review process.

Please submit your revised manuscript by May 19 2025 11:59PM. If you will need more time than this to complete your revisions, please reply to this message or contact the journal office at plosone@plos.org . When you're ready to submit your revision, log on to https://www.editorialmanager.com/pone/ and select the 'Submissions Needing Revision' folder to locate your manuscript file.

Please include the following items when submitting your revised manuscript:

- A rebuttal letter that responds to each point raised by the academic editor and reviewer(s). You should upload this letter as a separate file labeled 'Response to Reviewers'.
- A marked-up copy of your manuscript that highlights changes made to the original version. You should upload this as a separate file labeled 'Revised Manuscript with Track Changes'.
- An unmarked version of your revised paper without tracked changes. You should upload this as a separate file labeled 'Manuscript'.

If you would like to make changes to your financial disclosure, please include your updated statement in your cover letter. Guidelines for resubmitting your figure files are available below the reviewer comments at the end of this letter.

If applicable, we recommend that you deposit your laboratory protocols in protocols.io to enhance the reproducibility of your results. Protocols.io assigns your protocol its own identifier (DOI) so that it can be cited independently in the future. For instructions see: https://journals.plos.org/plosone/s/submission-guidelines#loc-laboratory-protocols . Additionally, PLOS ONE offers an option for publishing peer-reviewed Lab Protocol articles, which describe protocols hosted on protocols.io. Read more information on sharing protocols at https://plos.org/protocols?utm_medium=editorial-email&utm_source=authorletters&utm_campaign=protocols .

We look forward to receiving your revised manuscript.

Kind regards,

Zeheng Wang

Academic Editor

PLOS ONE

Journal Requirements:

Please review your reference list to ensure that it is complete and correct. If you have cited papers that have been retracted, please include the rationale for doing so in the manuscript text, or remove these references and replace them with relevant current references. Any changes to the reference list should be mentioned in the rebuttal letter that accompanies your revised manuscript. If you need to cite a retracted article, indicate the article’s retracted status in the References list and also include a citation and full reference for the retraction notice.

[Note: HTML markup is below. Please do not edit.]

Reviewers' comments:

Reviewer's Responses to Questions

**Comments to the Author**

1. If the authors have adequately addressed your comments raised in a previous round of review and you feel that this manuscript is now acceptable for publication, you may indicate that here to bypass the “Comments to the Author” section, enter your conflict of interest statement in the “Confidential to Editor” section, and submit your "Accept" recommendation.

Reviewer #1: All comments have been addressed

Reviewer #2: All comments have been addressed

Reviewer #3: All comments have been addressed

**********

2. Is the manuscript technically sound, and do the data support the conclusions?

The manuscript must describe a technically sound piece of scientific research with data that supports the conclusions. Experiments must have been conducted rigorously, with appropriate controls, replication, and sample sizes. The conclusions must be drawn appropriately based on the data presented. 

Reviewer #1: Yes

Reviewer #2: Yes

Reviewer #3: Yes

**********

3. Has the statistical analysis been performed appropriately and rigorously? 

Reviewer #1: Yes

Reviewer #2: Yes

Reviewer #3: Yes

**********

4. Have the authors made all data underlying the findings in their manuscript fully available?

The PLOS Data policy requires authors to make all data underlying the findings described in their manuscript fully available without restriction, with rare exception (please refer to the Data Availability Statement in the manuscript PDF file). The data should be provided as part of the manuscript or its supporting information, or deposited to a public repository. For example, in addition to summary statistics, the data points behind means, medians and variance measures should be available. If there are restrictions on publicly sharing data—e.g. participant privacy or use of data from a third party—those must be specified.

Reviewer #1: Yes

Reviewer #2: Yes

Reviewer #3: Yes

**********

5. Is the manuscript presented in an intelligible fashion and written in standard English?

PLOS ONE does not copyedit accepted manuscripts, so the language in submitted articles must be clear, correct, and unambiguous. Any typographical or grammatical errors should be corrected at revision, so please note any specific errors here.

Reviewer #1: Yes

Reviewer #2: Yes

Reviewer #3: Yes

**********

6. Review Comments to the Author

Please use the space provided to explain your answers to the questions above. You may also include additional comments for the author, including concerns about dual publication, research ethics, or publication ethics. (Please upload your review as an attachment if it exceeds 20,000 characters)

Reviewer #1: Thanks for inviting me to re-review the current manuscript. The authors clearly have carefully considered the comments provided, and took reasonable attempts to address the comments; which resulted a substantial improvement of this version of manuscript.

Particularly:

The inclusion of figure 4, table 5, and additional text describing the results on p.17-18, have provided valuable information for the readers in terms of how well the proposed three models in predicting DD. The authors have subsequently discussed the results and its implication in the discussion section, as well as drawing conclusion based on the current findings. Thus, this revised manuscript has provide valuable insight into this academic disciplines.

However, here are a few concerns that I recommend the authors to consider, to take the manuscript to a publishable state.

Major concern

Abstract:

I appreciate the new added text is to respond to Reviewer#2 1st comment, to provide some examples of therapy. However, currently it may create a confusion for the readers.

In objective: it mentioned 'we have investigated how the frequencies of three types of therapy, namely the physical therapy, the occupational therapy, and the speech therapy, received by a child can be exploited to predict whether the child suffers from DD or not'. And then the next sentence is '…The effectiveness of the proposed approach…'

These two sentences seem to imply the study aimed to investigate and compare the frequencies of three types of therapy predict DD. But in the method, results and conclusion sections, it mainly discussed different machine learning models.

Reading the manuscript, should the objective of the study is about the effectiveness of different machine learning based prediction models that predict DD in clinical settings? If it is, it seems that it is not reflected clearly in the objective section.

In the revised text, limitations section:

Line 315: 'Fourthly, due to the strict flowchart employed by the hospital, the outpatient medical records from which our dataset was derived are highly accurate and include minimal missing data and few unmeasured confounding variables.'

I found hard to understand why this is a limitation by reading the manuscript, especially in terms of 'highly accurate and minimal missing data'. If 'few unmeasured confounding variables' are the limitation, it would be great to have some elaboration, what they are (or could be), and how it may be a limitation. If 'highly accurate and minimal missing data' is actually a strength, suggest the author could explicitly acknowledging that it's the strength, or need not to mention.

Minor

Table 1, suggest improve consistency and readability of how data is reported, e.g., in number of samples:

Row 1: 'Total 876', row 2 '292 samples', later on '47 subjects', suggest to have more consistency; also some row reported overall sample size, and then break into different sub-group 292 Samples = 141 ASD+151

Non-ASD (4-11Yrs) , some only report sub-groups directly without overall N, '22 ASD Children, 16 Normal Children'

Suggest there should be abbreviations of all stat terms used in the table, e.g., NMI, DMN, AUC, etc.

Methods

Line 155, '…were previously given a diagnosis based on the criteria established in the…'

Can the author specify what diagnosis, e.g., DD? Or any diagnosis within DSM-IV TR, or developmental related Dx?

The use of the phrase 'suffer from DD' throughout the manuscript, while it may be valid; taking from a neuro-diversity affirming lens, would the authors open to consider using a more neutral word, e.g., 'will have DD, or will be diagnosed with DD'?

Thank you!

Reviewer #2: Thank you for your work. I truly appreciate the effort you put into addressing my concerns, and all of my questions have been resolved.

Reviewer #3: (No Response)

**********

7. PLOS authors have the option to publish the peer review history of their article (what does this mean? ). If published, this will include your full peer review and any attached files.

If you choose “no”, your identity will remain anonymous but your review may still be made public.

**Do you want your identity to be public for this peer review?** For information about this choice, including consent withdrawal, please see our Privacy Policy .

Reviewer #1: No

Reviewer #2: No

Reviewer #3: **Yes: ** Xianwei Zhang

**********

[NOTE: If reviewer comments were submitted as an attachment file, they will be attached to this email and accessible via the submission site. Please log into your account, locate the manuscript record, and check for the action link "View Attachments". If this link does not appear, there are no attachment files.]

While revising your submission, please upload your figure files to the Preflight Analysis and Conversion Engine (PACE) digital diagnostic tool, https://pacev2.apexcovantage.com/ . PACE helps ensure that figures meet PLOS requirements. To use PACE, you must first register as a user. Registration is free. Then, login and navigate to the UPLOAD tab, where you will find detailed instructions on how to use the tool. If you encounter any issues or have any questions when using PACE, please email PLOS at figures@plos.org . Please note that Supporting Information files do not need this step.

# Author comment: Author response to Decision Letter 1

**DOI:** 10.1371/journal.pone.0324204.r005

17 Apr 2025

Comment 1 from reviewer 1:

The authors clearly have carefully considered the comments provided, and took reasonable attempts to address the comments; which resulted a substantial improvement of this version of manuscript.

Particularly:

The inclusion of figure 4, table 5, and additional text describing the results on p.17-18, have provided valuable information for the readers in terms of how well the proposed three models in predicting DD. The authors have subsequently discussed the results and its implication in the discussion section, as well as drawing conclusion based on the current findings. Thus, this revised manuscript has provide valuable insight into this academic disciplines.

Response:

Thank you very much for your positive comments.

[ Major concern ]

Comment 2 from reviewer 1:

I appreciate the new added text is to respond to Reviewer#2 1st comment, to provide some examples of therapy. However, currently it may create a confusion for the readers.

In objective: it mentioned 'we have investigated how the frequencies of three types of therapy, namely the physical therapy, the occupational therapy, and the speech therapy, received by a child can be exploited to predict whether the child suffers from DD or not'. And then the next sentence is '…The effectiveness of the proposed approach…'

These two sentences seem to imply the study aimed to investigate and compare the frequencies of three types of therapy predict DD. But in the method, results and conclusion sections, it mainly discussed different machine learning models.

Reading the manuscript, should the objective of the study is about the effectiveness of different machine learning based prediction models that predict DD in clinical settings? If it is, it seems that it is not reflected clearly in the objective section.

Response:

In this study, we have aimed to investigated how the frequencies of three types of therapy, namely the physical therapy, the occupational therapy, and the speech therapy, received by a child can be exploited to predict whether the child suffers from DD or not. The reason why we built alternative categories of prediction models was to investigate how the DT models performed in our applications in comparison with advanced machine learning models such as the SVM models and the DNN models. As the users of DT models can easily interpret how the predictions are made by examining the explicit decision rules output by the algorithm, the DT models are favorite for applications in which the DT models can deliver the same level of performance as advanced machine learning models. In order to clarify our objective, we have added the following statements to the first paragraph of section “Development of prediction models and performance evaluation” (lines 229-234): Therefore, it is of interest to investigate how the performance of alternative categories of machine learning models compares. If the performance of the DT models observed in the experiments is comparable with the performance of the advanced machine learning models, which was observed in our recent studies [43-44], then the DT models are favorite due to explicit decision rules output by the algorithm.

Furthermore, we have added the following statement to the second paragraph of section “Discussion” (lines 306-307):

Fortunately, for our applications, we do not need to trade performance for the interpretability of the prediction model.

Comment 3 from reviewer 1:

In the revised text, limitations section:

Line 315: 'Fourthly, due to the strict flowchart employed by the hospital, the outpatient medical records from which our dataset was derived are highly accurate and include minimal missing data and few unmeasured confounding variables.'

I found hard to understand why this is a limitation by reading the manuscript, especially in terms of 'highly accurate and minimal missing data'. If 'few unmeasured confounding variables' are the limitation, it would be great to have some elaboration, what they are (or could be), and how it may be a limitation. If 'highly accurate and minimal missing data' is actually a strength, suggest the author could explicitly acknowledging that it's the strength, or need not to mention.

Response:

We apologize for the confusion. We have deleted this statement from the first paragraph of section “Discussion”. Instead, we added the following statement to the second paragraph of section “Data collection and outcome measurement

”(lines 191-193):

It is observed that due to the strict flowchart employed by the hospital, the outpatient medical records from which our dataset was derived are highly accurate and include minimal missing data and few unmeasured confounding variables.

[ Minor concern ]

Comment 4 from reviewer 1:

Table 1, suggest improve consistency and readability of how data is reported, e.g., in number of samples:

Row 1: 'Total 876', row 2 '292 samples', later on '47 subjects', suggest to have more consistency; also some row reported overall sample size, and then break into different sub-group 292 Samples = 141 ASD+151

Non-ASD (4-11Yrs) , some only report sub-groups directly without overall N, '22 ASD Children, 16 Normal Children'

Suggest there should be abbreviations of all stat terms used in the table, e.g., NMI, DMN, AUC, etc.

Response:

Thank you for your suggestions. We have added abbreviations for all statistical terms used in the table and revised the description of sample sizes to enhance data clarity and readability. (lines 136-143)

Comment 5 from reviewer 1:

Methods

Line 155, '…were previously given a diagnosis based on the criteria established in the…'

Can the author specify what diagnosis, e.g., DD? Or any diagnosis within DSM-IV TR, or developmental related Dx?

Response:

Thank you for your feedback. Accordingly, we have included the following statement: “For example, the DSM-5-TR defines autism spectrum disorder (ASD) as involving persistent deficits in social communication across multiple environments, as outlined in the relevant diagnostic criterion” (lines 162–164)

Comment 6 from reviewer 1:

The use of the phrase 'suffer from DD' throughout the manuscript, while it may be valid; taking from a neuro-diversity affirming lens, would the authors open to consider using a more neutral word, e.g., 'will have DD, or will be diagnosed with DD'?

Response:

Thank you for your suggestions. We have replaced the phrase 'suffer from DD' with 'will/may develop DD' throughout the manuscript to adopt more neutral and objective language.

Attachment Submitted filename: Response to Reviewers-20250416.docx

# Editor report: Decision Letter 2

**DOI:** 10.1371/journal.pone.0324204.r006

**Authors:** Zeheng Wang

22 Apr 2025

Detection of Pediatric Developmental Delay with Machine Learning Technologies

PONE-D-24-35849R2

Dear Dr. Oyang,

We’re pleased to inform you that your manuscript has been judged scientifically suitable for publication and will be formally accepted for publication once it meets all outstanding technical requirements.

Within one week, you’ll receive an e-mail detailing the required amendments. When these have been addressed, you’ll receive a formal acceptance letter and your manuscript will be scheduled for publication.

An invoice will be generated when your article is formally accepted. Please note, if your institution has a publishing partnership with PLOS and your article meets the relevant criteria, all or part of your publication costs will be covered. Please make sure your user information is up-to-date by logging into Editorial Manager at Editorial Manager®  and clicking the ‘Update My Information' link at the top of the page. If you have any questions relating to publication charges, please contact our Author Billing department directly at authorbilling@plos.org.

If your institution or institutions have a press office, please notify them about your upcoming paper to help maximize its impact. If they’ll be preparing press materials, please inform our press team as soon as possible -- no later than 48 hours after receiving the formal acceptance. Your manuscript will remain under strict press embargo until 2 pm Eastern Time on the date of publication. For more information, please contact onepress@plos.org.

Kind regards,

Zeheng Wang

Academic Editor

PLOS ONE

Additional Editor Comments (optional):

Reviewers' comments:

# Editor report: Acceptance letter

**DOI:** 10.1371/journal.pone.0324204.r007

**Authors:** Zeheng Wang

PONE-D-24-35849R2

PLOS ONE

Dear Dr. Oyang,

I'm pleased to inform you that your manuscript has been deemed suitable for publication in PLOS ONE. Congratulations! Your manuscript is now being handed over to our production team.

At this stage, our production department will prepare your paper for publication. This includes ensuring the following:

* All references, tables, and figures are properly cited

* All relevant supporting information is included in the manuscript submission,

* There are no issues that prevent the paper from being properly typeset

You will receive further instructions from the production team, including instructions on how to review your proof when it is ready. Please keep in mind that we are working through a large volume of accepted articles, so please give us a few days to review your paper and let you know the next and final steps.

Lastly, if your institution or institutions have a press office, please let them know about your upcoming paper now to help maximize its impact. If they'll be preparing press materials, please inform our press team within the next 48 hours. Your manuscript will remain under strict press embargo until 2 pm Eastern Time on the date of publication. For more information, please contact onepress@plos.org.

If we can help with anything else, please email us at customercare@plos.org.

Thank you for submitting your work to PLOS ONE and supporting open access.

Kind regards,

PLOS ONE Editorial Office Staff

on behalf of

Dr. Zeheng Wang

Academic Editor

PLOS ONE
